# Supplementary material for: Synaptic connectome of the Drosophila circadian clock
Source: Nat Commun. 2024 Dec 5;15:10392. doi: 10.1038/s41467-024-54694-0 (PMC11621569; doi:10.1038/s41467-024-54694-0)
Supplement: Supplementary file 1 — Supplementary Information [file 41467_2024_54694_MOESM1_ESM.pdf]

Synaptic connectome of the *Drosophila* circadian clock

Supplementary Information

Reinhard and Fukuda *et al.* 2024

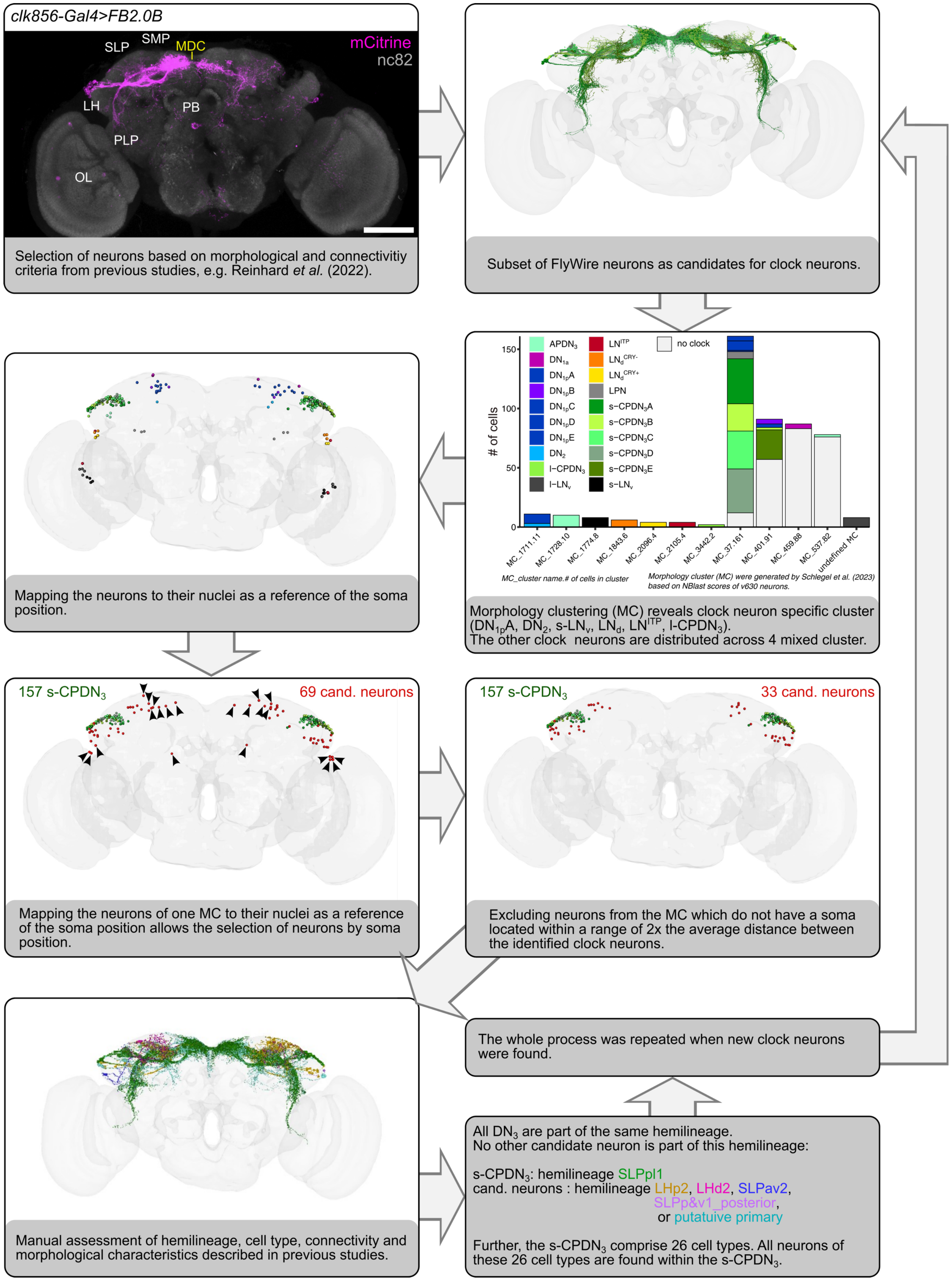
clk856-Gal4>FB2.0B
SLP
SMP
MDC
mCitrine
nc82
LH
PB
PLP
OL
Selection of neurons based on morphological and connectivitiy criteria from previous studies, e.g. Reinhard et al. (2022).
Subset of FlyWire neurons as candidates for clock neurons.
APDN$_3$
DN$_{1a}$
DN$_{1p}$A
DN$_{1p}$B
DN$_{1p}$C
DN$_{1p}$D
DN$_{1p}$E
DN$_2$
l-CPDN$_3$
l-LN$_v$
LN^{ITP}
LN$_d^{CRY-}$
LN$_d^{CRY+}$
LPN
s-CPDN$_3$A
s-CPDN$_3$B
s-CPDN$_3$C
s-CPDN$_3$D
s-CPDN$_3$E
s-LN$_v$
no clock
# of cells
150
100
50
0
MC_1711.11
MC_1728.10
MC_1774.8
MC_1843.6
MC_2096.4
MC_2105.4
MC_3442.2
MC_37.161
MC_401.91
MC_459.88
MC_537.82
undefined MC
MC_cluster name.# of cells in cluster
Morphology cluster (MC) were generated by Schlegel et al. (2023) based on NBlast scores of v630 neurons.
Morphology clustering (MC) reveals clock neuron specific cluster (DN$_{1p}$A, DN$_2$, s-LN$_v$, LN$_d$, LN^{ITP}, l-CPDN$_3$).
The other clock neurons are distributed across 4 mixed cluster.
Mapping the neurons to their nuclei as a reference of the soma position.
157 s-CPDN$_3$
69 cand. neurons
Mapping the neurons of one MC to their nuclei as a reference of the soma position allows the selection of neurons by soma position.
157 s-CPDN$_3$
33 cand. neurons
Excluding neurons from the MC which do not have a soma located within a range of 2x the average distance between the identified clock neurons.
The whole process was repeated when new clock neurons were found.
Manual assessment of hemilineage, cell type, connectivity and morphological characteristics described in previous studies.
All DN$_3$ are part of the same hemilineage.
No other candidate neuron is part of this hemilineage:
s-CPDN$_3$: hemilineage SLPpl1
cand. neurons : hemilineage LHp2, LHd2, SLPav2, SLPp&v1_posterior, or putatuive primary
Further, the s-CPDN$_3$ comprise 26 cell types. All neurons of these 26 cell types are found within the s-CPDN$_3$.

Supplementary Fig. 1: Pipeline for identifying clock neurons in the FlyWire dataset. Neurons were identified based on morphological or connectivity features described previously [1, 2, 3, 4, 5] or based on NBLAST similarity to identified clock neurons in the hemibrain [6]. Morphology clustering (v630, [6]) was then used to identify neurons with similar morphology. Several clock neurons formed clock neuron-specific morphology clusters (e.g. $APDN_3$, s-LN_v, and LN^{ITP} amongst others). If clock neurons did not form a unique morphology cluster, all neurons of the corresponding morphology cluster were considered as possible candidates. The cell body position was determined by mapping the coordinates of the nuclei [7] to the root ids of the neurons. The average distance between the cell body position of neurons in one clock cluster was determined per hemisphere and candidate neurons laying within twice the average distance (neurons laying further apart are marked by arrowheads) were manually assessed based on morphological features and connectivity described in previous studies. In addition, hemilineage and cell type information was used to determine whether the candidate neurons could be additional clock neurons. If new clock neurons were identified, the whole procedure was repeated. All identified clock neurons include all neurons of the different cell types found within these clock neurons. By definition, a cell type is a uniquely identifiable neuron in the dataset [6]. All numbers refer to neurons across both hemispheres. Source data are provided in the Source Data file.

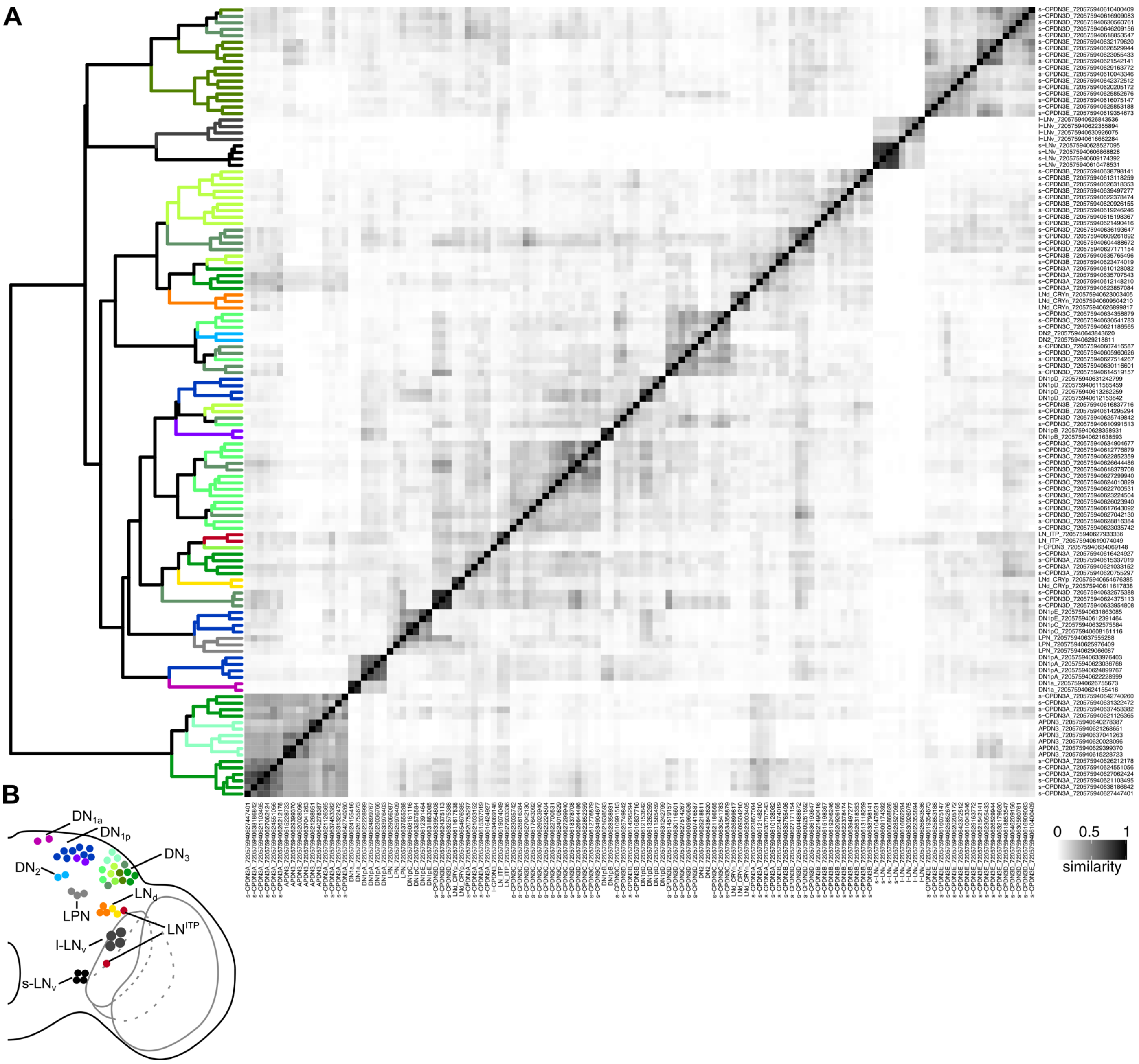

Supplementary Fig. 2: Clustering of clock neurons based on their total inputs and outputs. (A) Cosine similarity matrix of clock neurons of the right hemisphere based on their total inputs and outputs from other neurons. For the similarity analysis, no threshold for the number of synapses was used. The darker the color, the higher the similarity between neurons. Neurons within the clades are colored based on the schematic in **(B).** Except for DN_3 and DN_{1p}, all neurons from one clock neuron group cluster together. While some DN_3 form DN_3-specific clusters, other DN_3 often also cluster with other clock neuron groups. Source data for panel A are provided in the Source Data file.

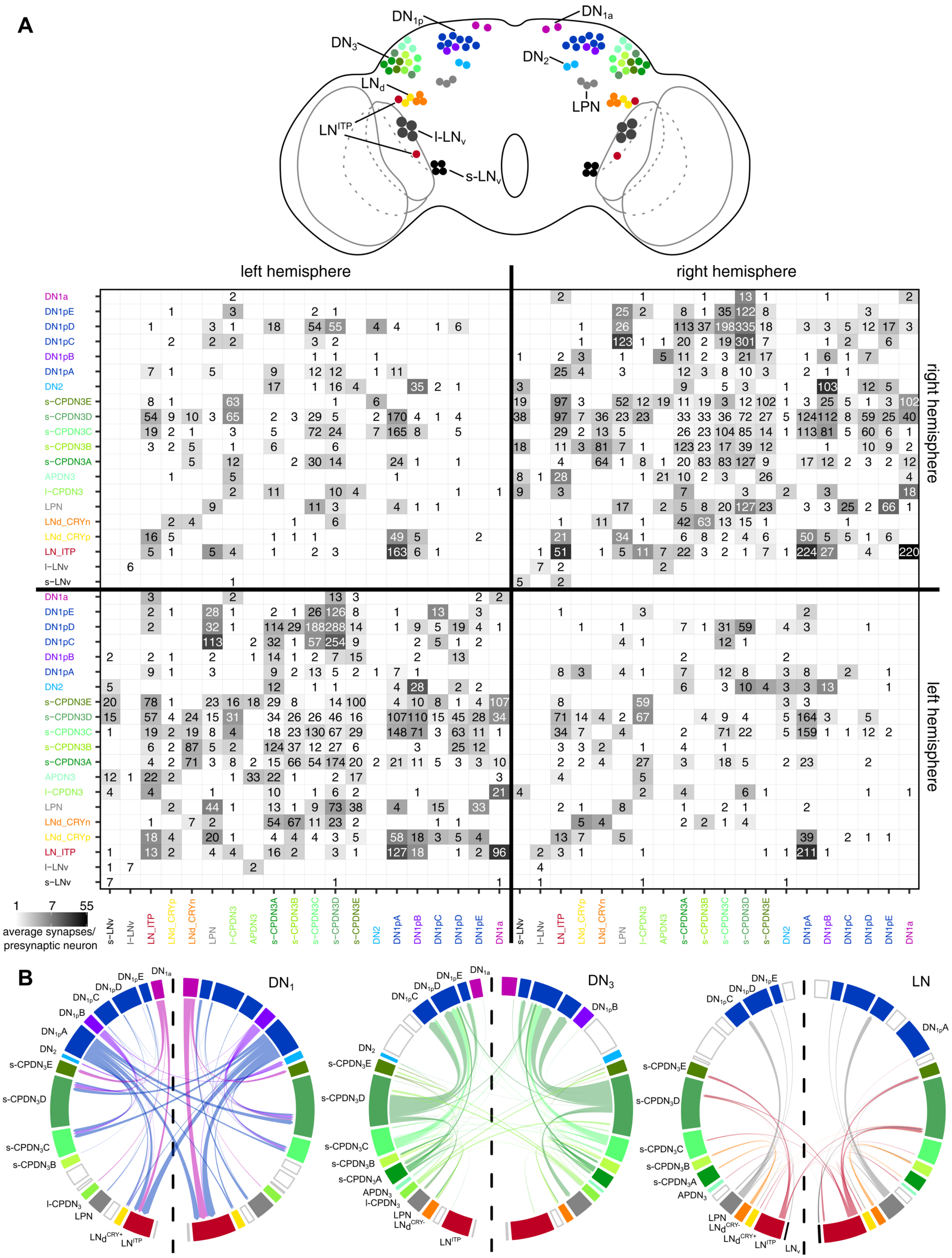

Supplementary Fig. 3: Connectivity between different clock clusters. (A) Connectivity matrix highlighting the interconnectivity between different clock cell types across the two hemispheres. Locations of different clock cells are depicted in the schematic. The numbers within the matrix indicate the number of synapses. No synapse threshold was applied. Grey shading reflects the average strength of the connections. **(B)** Clock interconnectivity as shown in Figure 1 separated by DN_1, DN_3, and LN. Only connections greater than 4 synapses were considered. Source data are provided in the Source Data file.

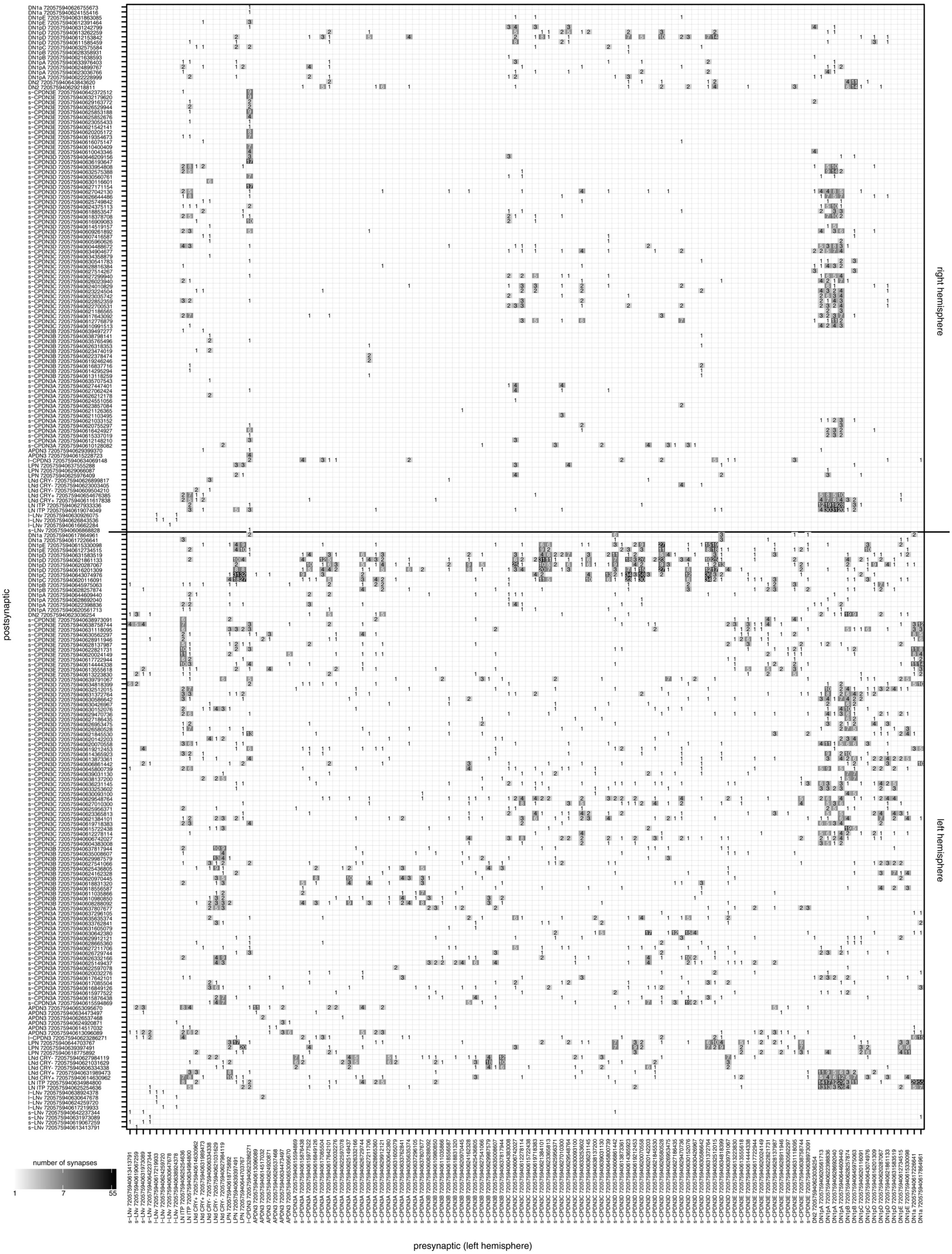

Supplementary Fig. 4: Connectivity from individual clock cells in the left hemisphere to all clock cells. Connectivity matrix highlighting the interconnectivity between individual clock cells. Grey shading and the numbers within the matrix indicate the number of synapses per connection. Black indicates strong connectivity and white indicates low or no connectivity strength. No synapse threshold was applied.

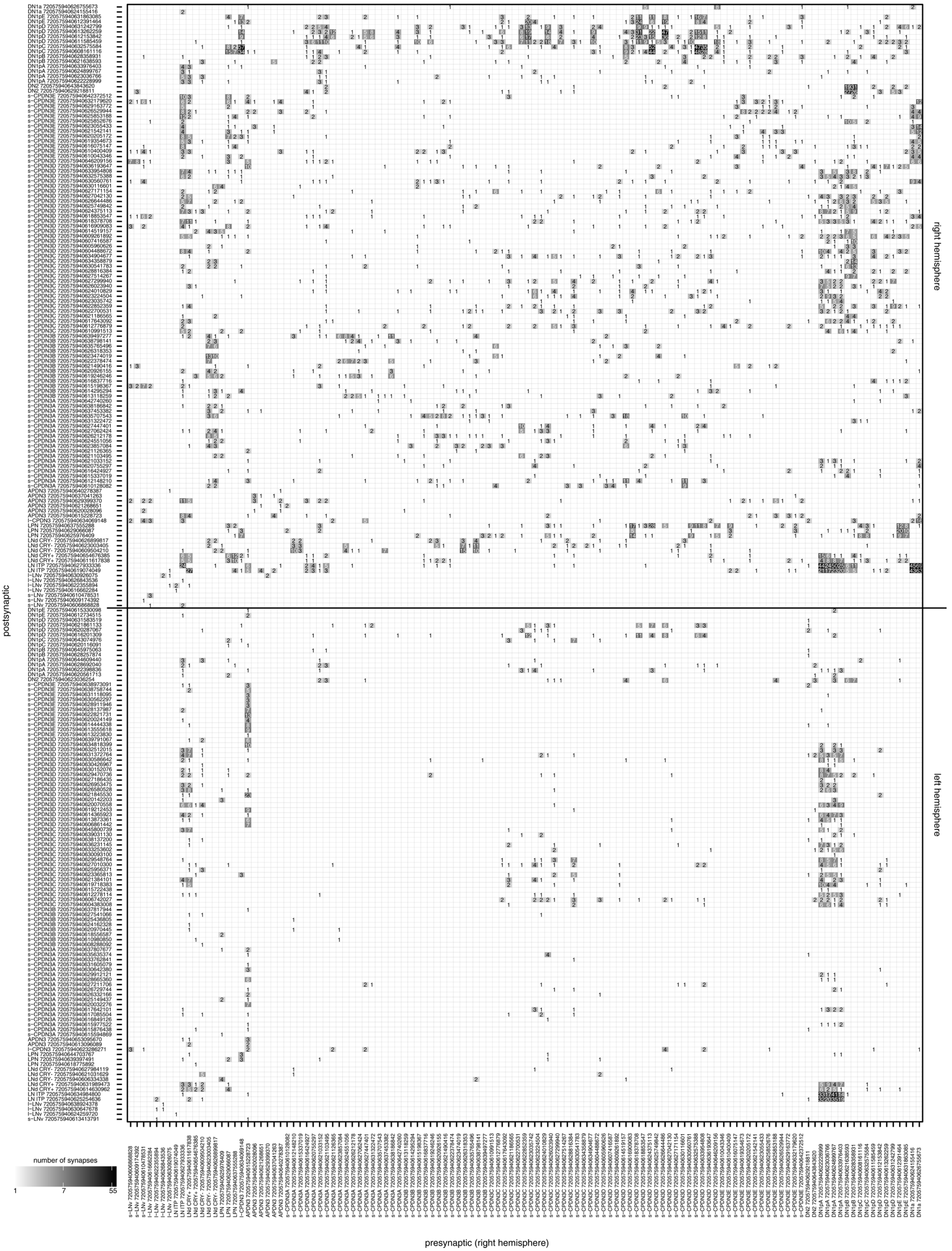

Supplementary Fig. 5: Connectivity from individual clock cells in the right hemisphere to all clock cells. Connectivity matrix highlighting the interconnectivity between individual clock cells. Grey shading and the numbers within the matrix indicate the number of synapses per connection. Black indicates strong connectivity and white indicates low or no connectivity strength. No synapse threshold was applied.

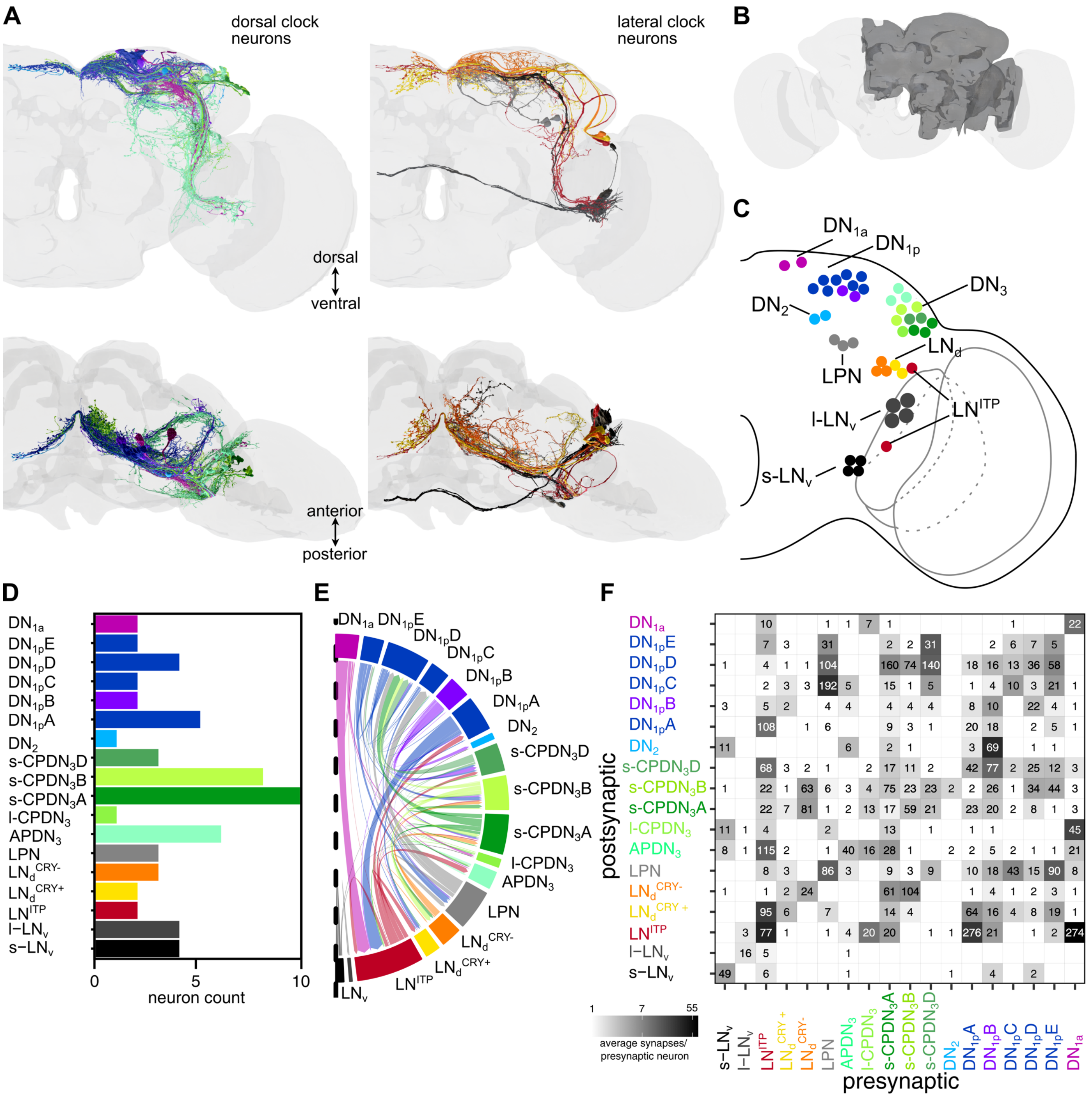

Supplementary Fig. 6: *Drosophila* hemibrain circadian clock network. (A) Reconstructions of identified clock neurons in the **(B)** *Drosophila* hemibrain connectome**. (C)** Schematic showing the locations of different clock neuron cell types. **(D)** Numbers of the different clock cell types that were identified in the hemibrain connectome. **(E)** Synaptic interconnectivity between different cell types within the clock network. The direction of the arrow indicates the flow of information. Only connections with >9 synapses were taken into account. The dashed line indicates the brain midline. **(F)** Connectivity matrix highlighting the interconnectivity between different clock cell types. The numbers within the matrix indicate the number of synapses. Grey shading reflects the average strength of this connectivity. No synapse threshold was applied. Source data for panels D-F are provided in the Source Data file.

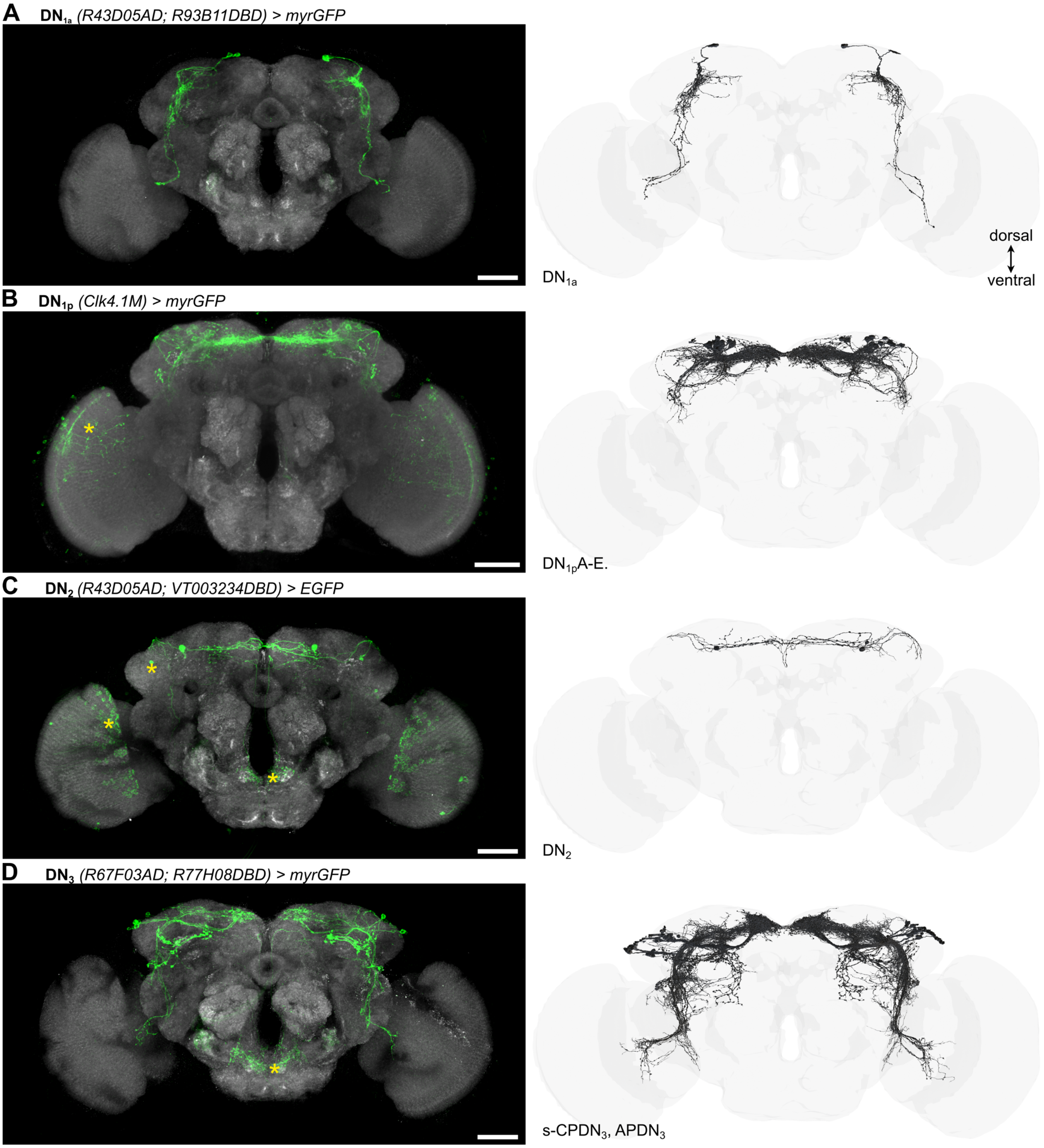

Supplementary Fig. 7: Expression patterns of Gal4 lines targeting different populations of DN. Confocal stacks showing GFP expression in **(A)** DN_{1a}, **(B)** DN_{1p}, **(C)** DN_2, and **(D)** DN_3 that are included in the different Gal4 lines. Reconstructions of corresponding neurons in the FlyWire connectome are provided on the right. Neurons not part of the clock network are marked by an asterisk. Scale bars = 50μm.

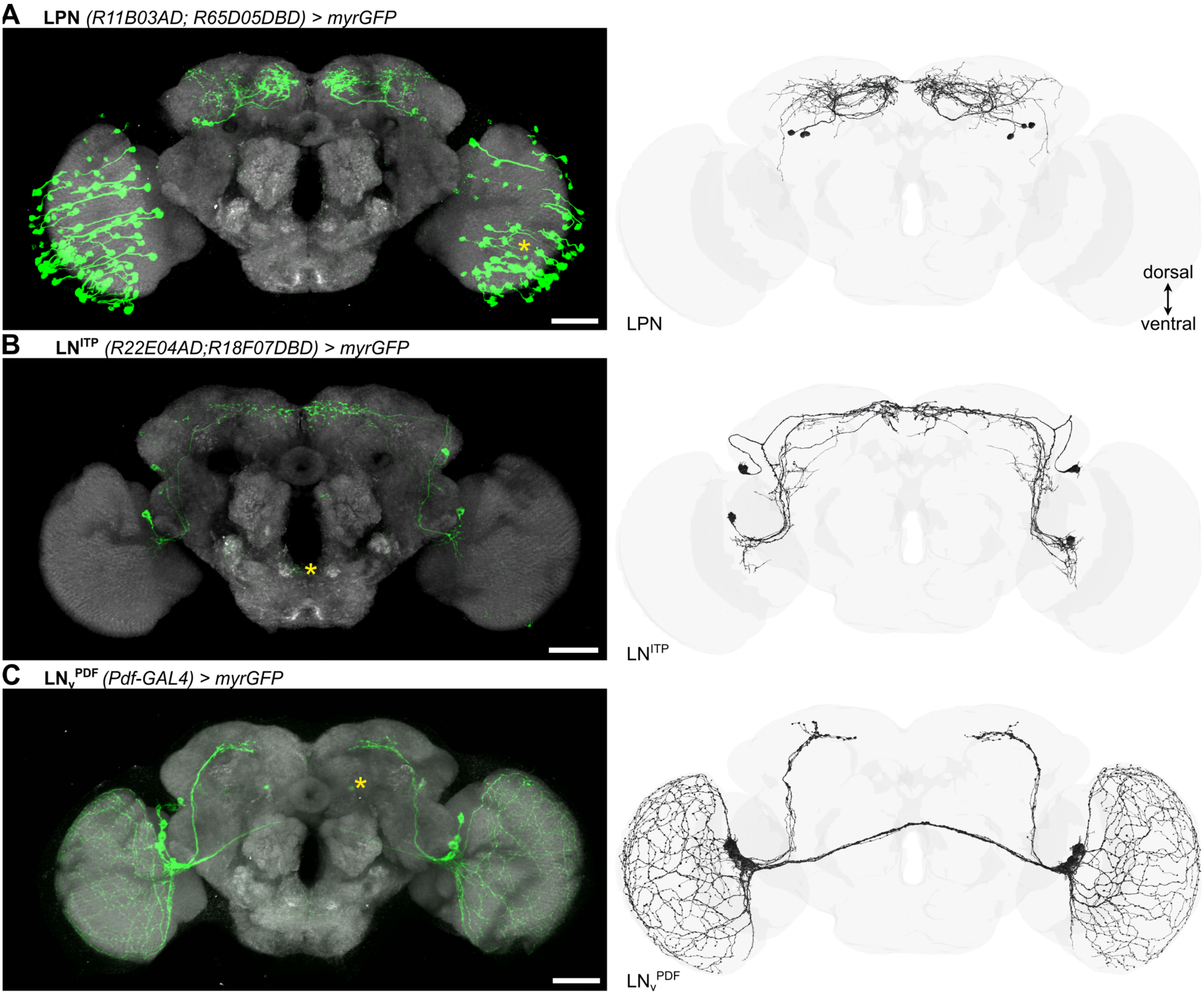

Supplementary Fig. 8: Expression patterns of Gal4 lines targeting different populations of LN. Confocal stacks showing GFP expression in **(A)** LPN, **(B)** LN^{ITP}, and **(C)** LN_V^{PDF} that are included in the different Gal4 lines. Reconstructions of corresponding neurons in the FlyWire connectome are provided on the right. Neurons not part of the clock network are marked by an asterisk. Scale bars = 50µm.

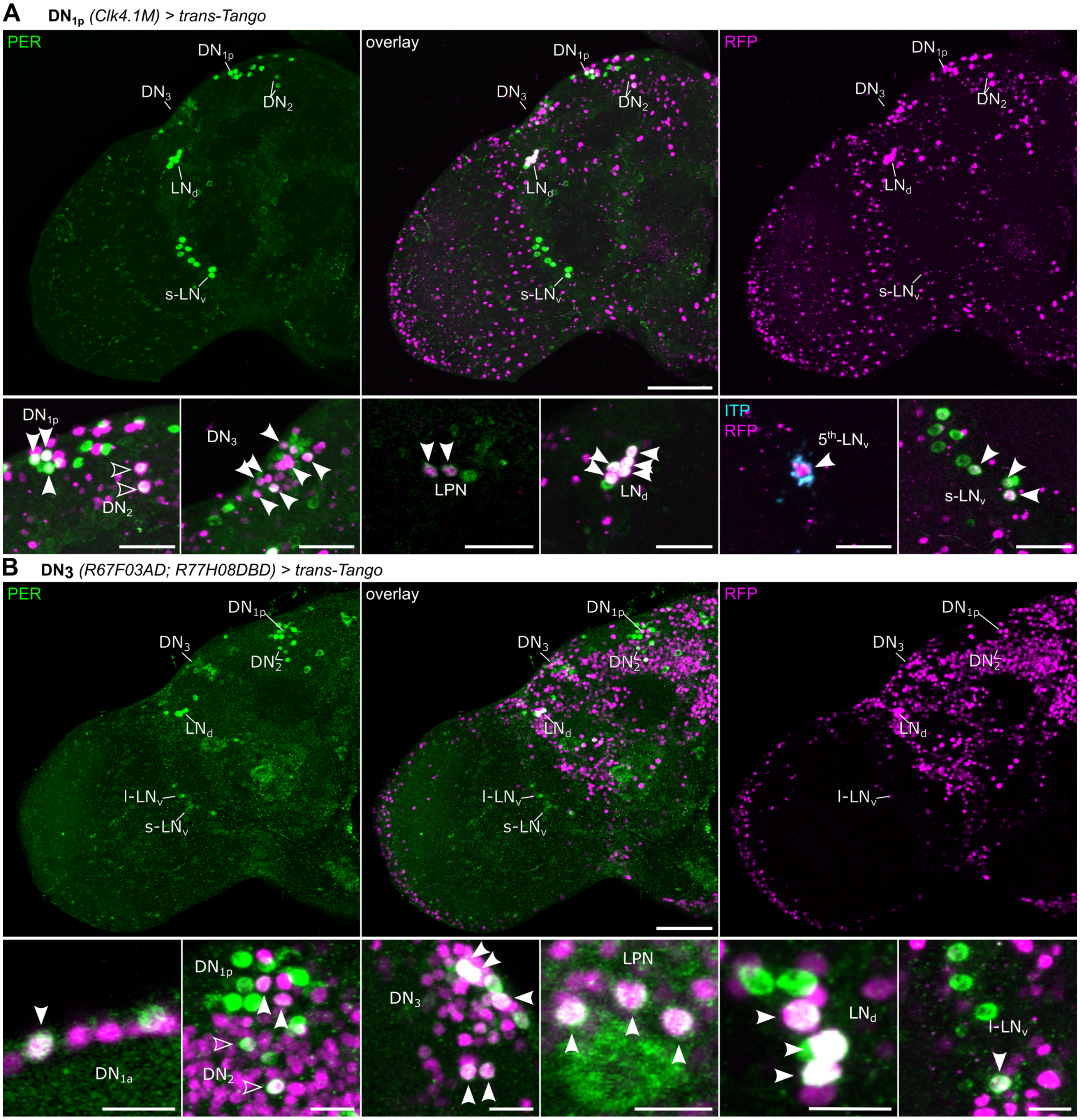

Supplementary Fig. 9: Validating connectivity within the clock network using *trans*-Tango. (A) Driving *trans*-Tango using *Clk4.1M-Gal4* revealed DN_{1p}, DN_2, DN_3, LPN, LN_d, 5th-LN_v, and s-LN_v as post-synaptic partners of DN_{1p}. **(B)** Expressing *trans*-Tango in a subset of the DN_3 (including s-CPDN_3 and APDN_3) generated a post-synaptic signal in all dorsal clock neuron clusters, as well as LPN, LN_d, and l-LN_v. Scale bars = 50µm for overview and 20µm for higher magnification images. These two examples highlight a general agreement in synaptic connectivity observed with the connectomes and *trans*-Tango. Abbreviations: PER, Period; ITP, Ion transport peptide; RFP, red fluorescent protein.

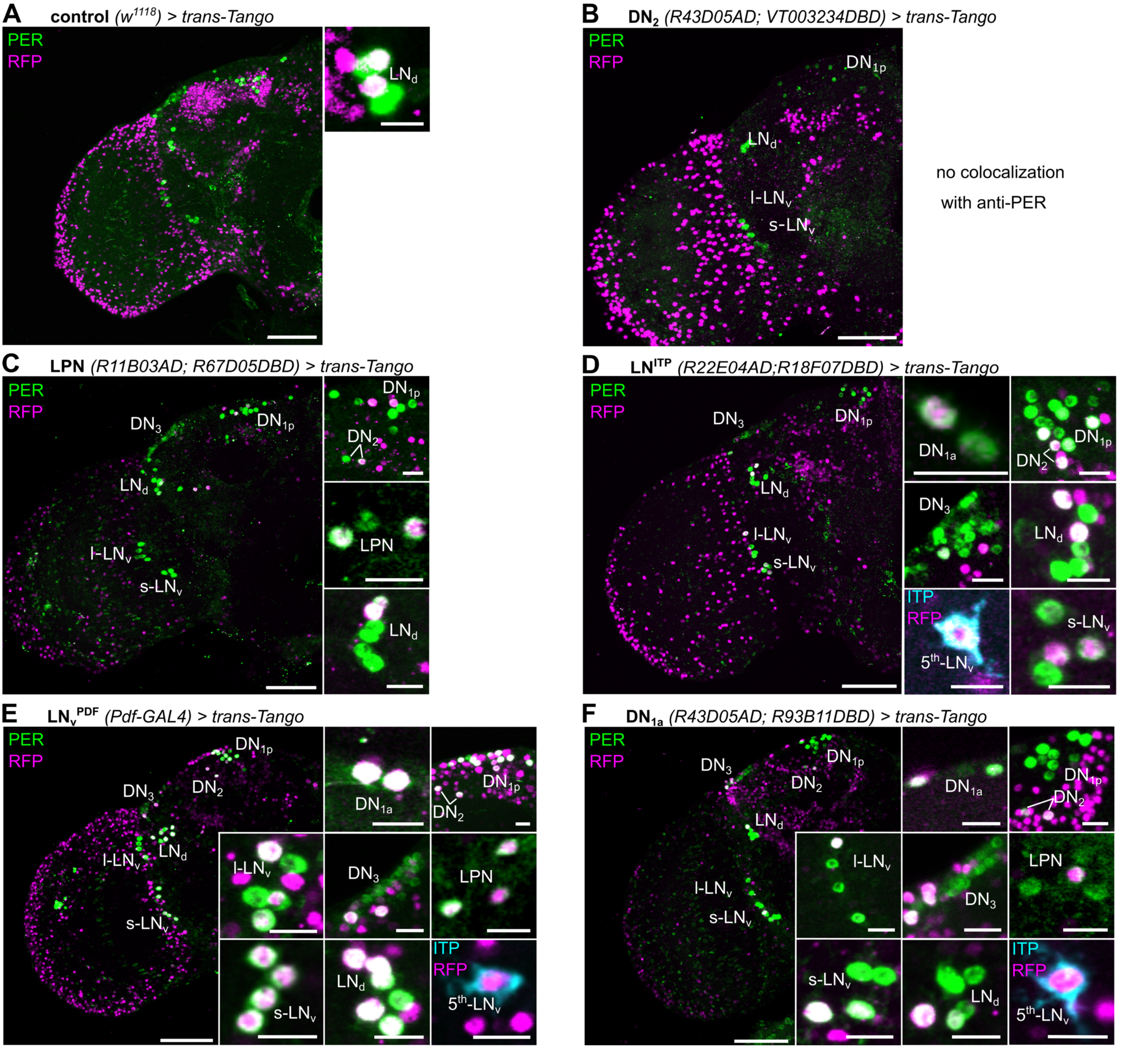

Supplementary Fig. 10: Validating connectivity within the clock network using *trans*-Tango. (A) Control for *trans*-Tango occasionally generated a false post-synaptic signal in two LN$_d$. Expressing *trans*-Tango in **(B)** DN$_2$ does not generate a post-synaptic signal in any clock neurons but **(C)** LPN, **(D)** LN^{ITP}, **(E)** LN$_V^{PDF,}$ and **(F)** DN$_{1a}$ are all presynaptic to other clock neurons. Scale bars = 50µm for overview and 20µm for higher magnification images. Abbreviations: PER, Period; ITP, Ion Transport Peptide; RFP, Red Fluorescent Protein.

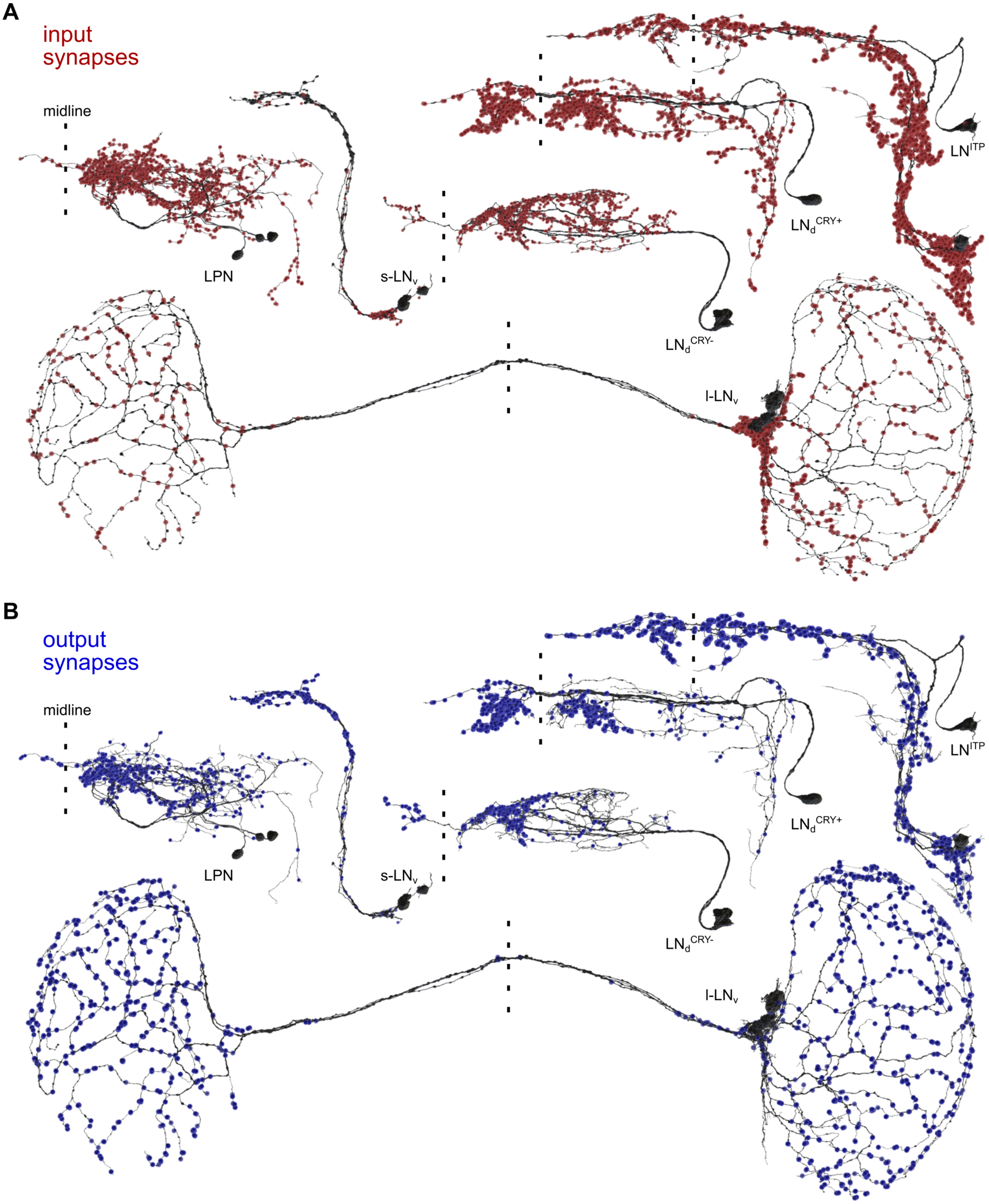

Supplementary Fig. 11: Synapse locations of lateral clock neurons. (A) Postsynaptic (red) and **(B)** presynaptic (blue) sites of different groups of lateral clock neurons in the right hemisphere. The dashed line indicates the brain midline. Distinguishable dendrites are seen seldomly.

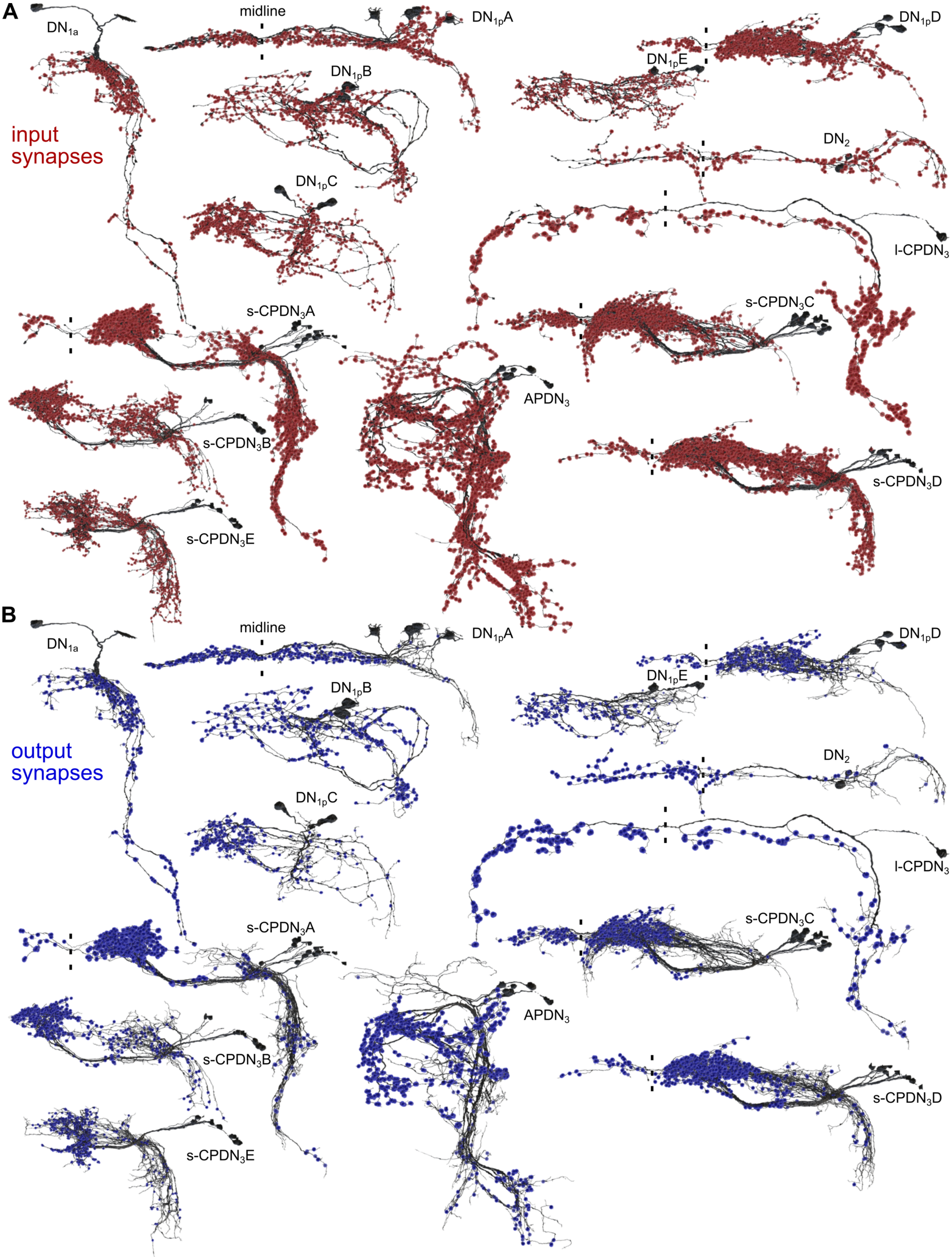

Supplementary Fig. 12: Synapse locations of dorsal clock neurons. (A) Postsynaptic (red) and **(B)** presynaptic (blue) sites of different groups of dorsal clock neurons in the right hemisphere. The dashed line indicates the brain midline. Distinguishable dendrites are seen seldomly.

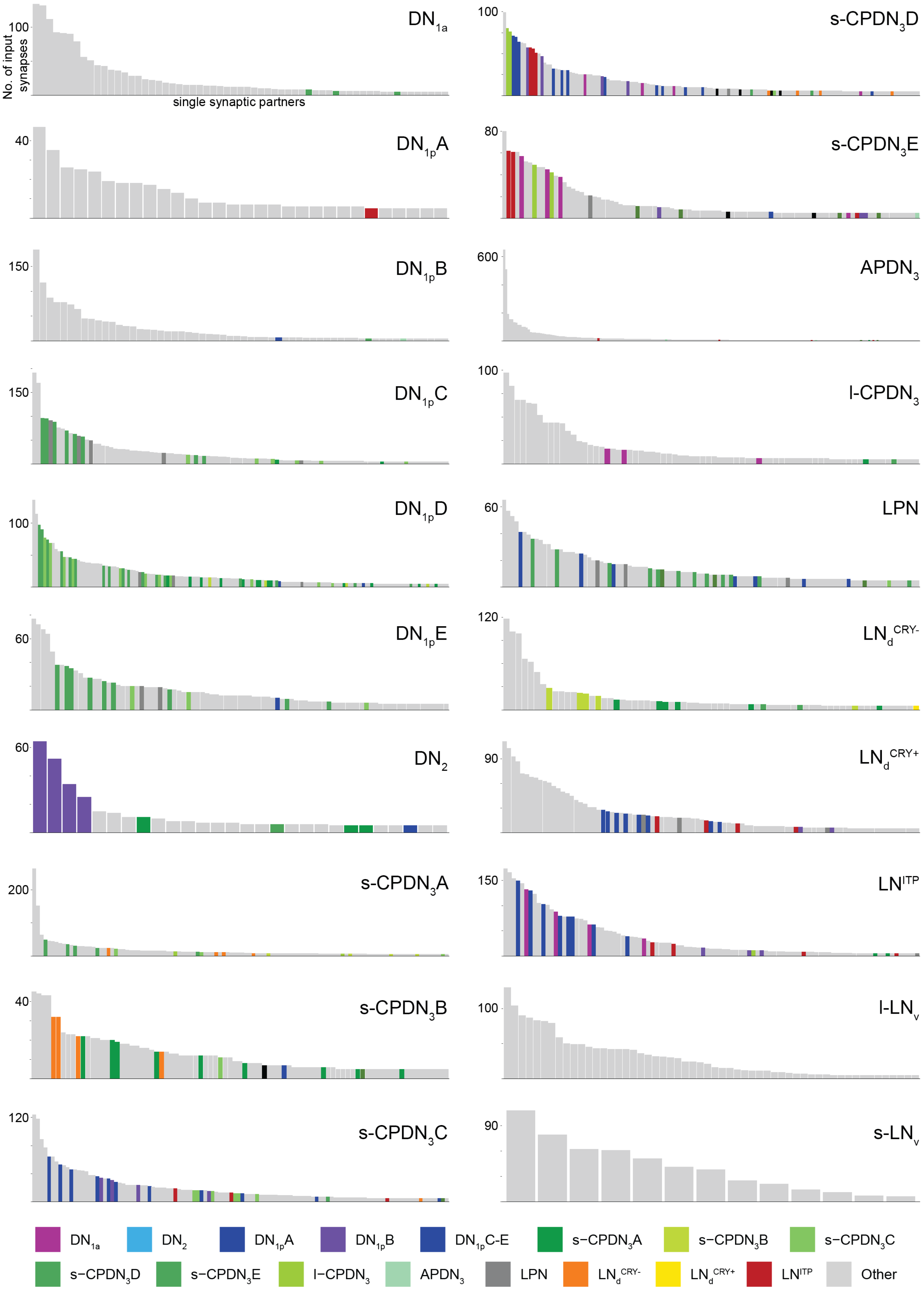

Supplementary Fig. 13: Individual presynaptic partners of clock neurons. Individual presynaptic partners of different clock neurons are sorted based on the number of synapses. Presynaptic clock neurons are colored based on their identity. All inputs across both hemispheres are shown. A threshold of 5 synapses was used for connections to be considered valid. Source data are provided in the Source Data file.

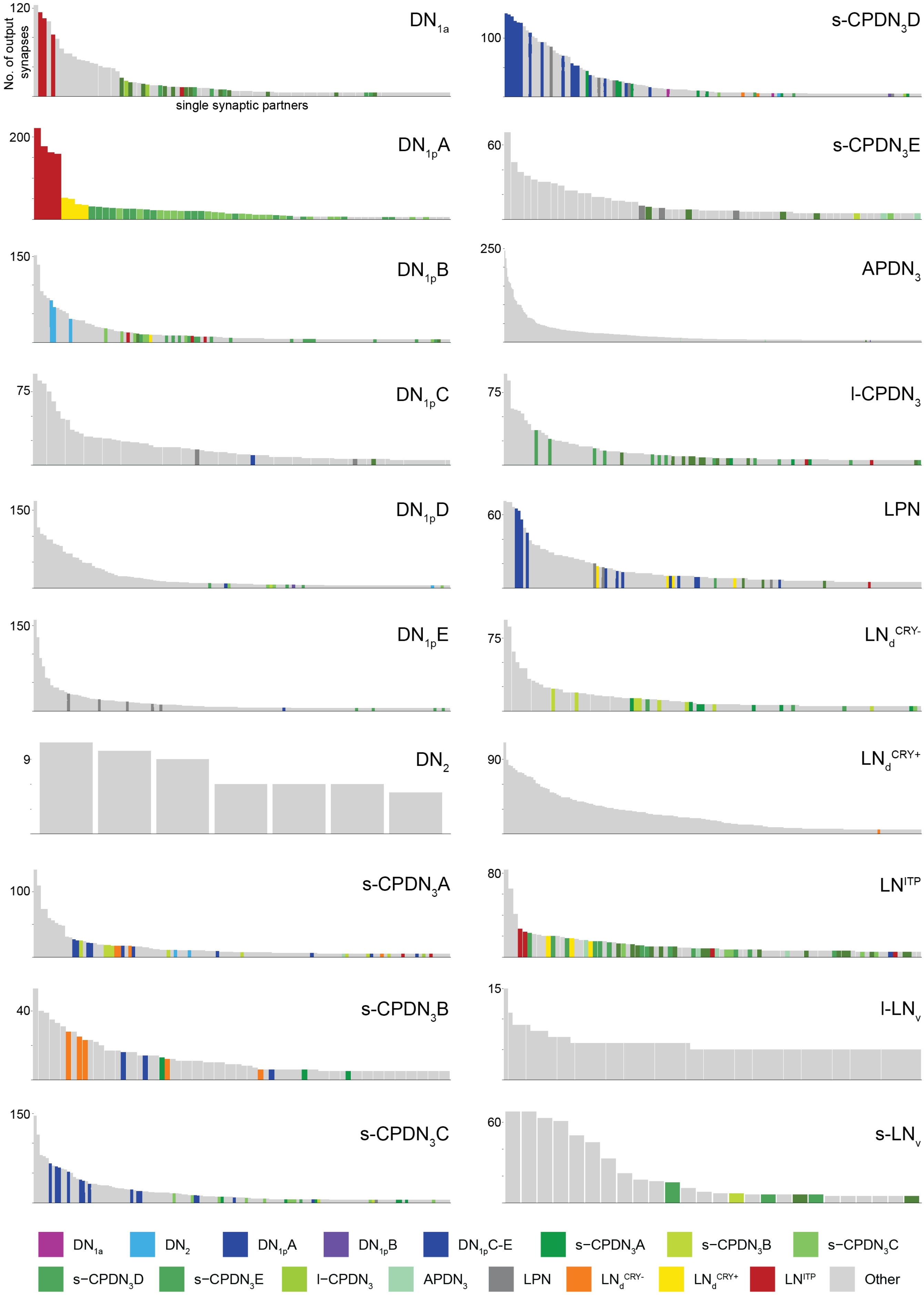

Supplementary Fig. 14: Individual postsynaptic partners of clock neurons. Individual postsynaptic partners of different clock neurons are sorted based on the number of synapses. Postsynaptic clock neurons are colored based on their identity. All inputs across both hemispheres are shown. A threshold of 5 synapses was used for connections to be considered valid. Source data are provided in the Source Data file.

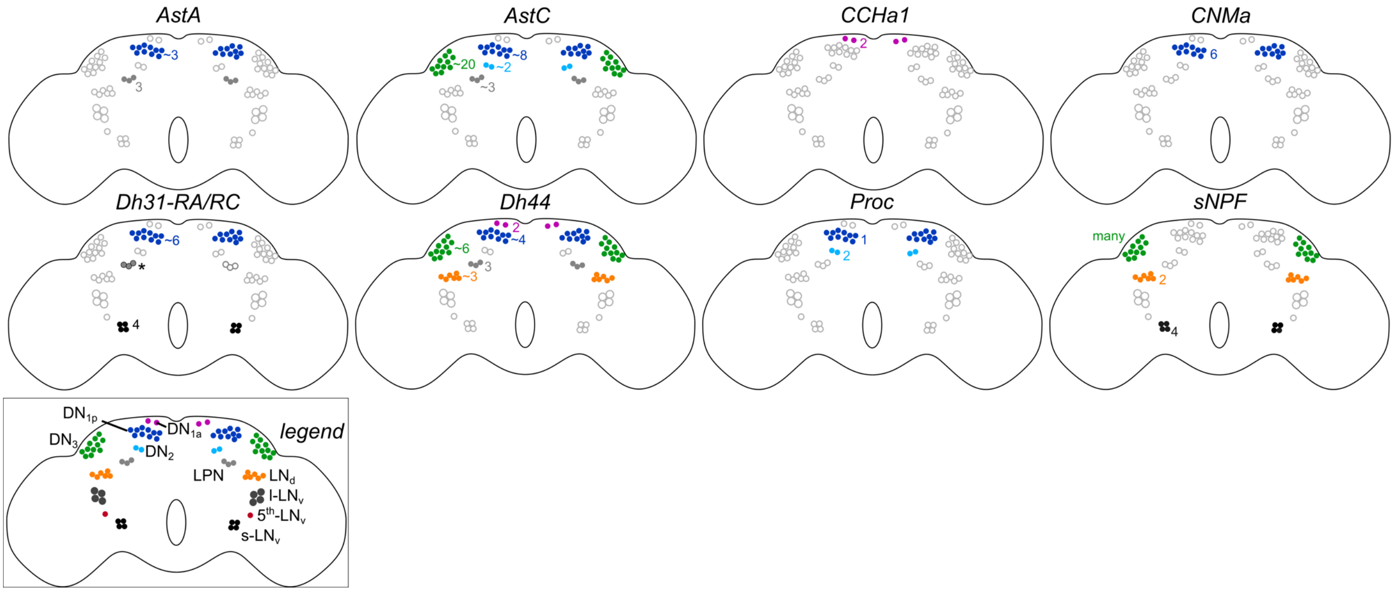

Supplementary Fig. 15: Neuropeptide expression in clock neurons. Schematics showing the clock neurons that express different neuropeptides. Schematics are based on expression mapping reported in Supplementary Data 3. The numbers of clock cells of a given type that express the neuropeptide are also included where appropriate. DH31 expression schematic is based on GFP expression in Supplementary Data 3 and antibody staining reported earlier (marked by an asterisk) [2]. Numbers refer to the expression in one hemisphere.

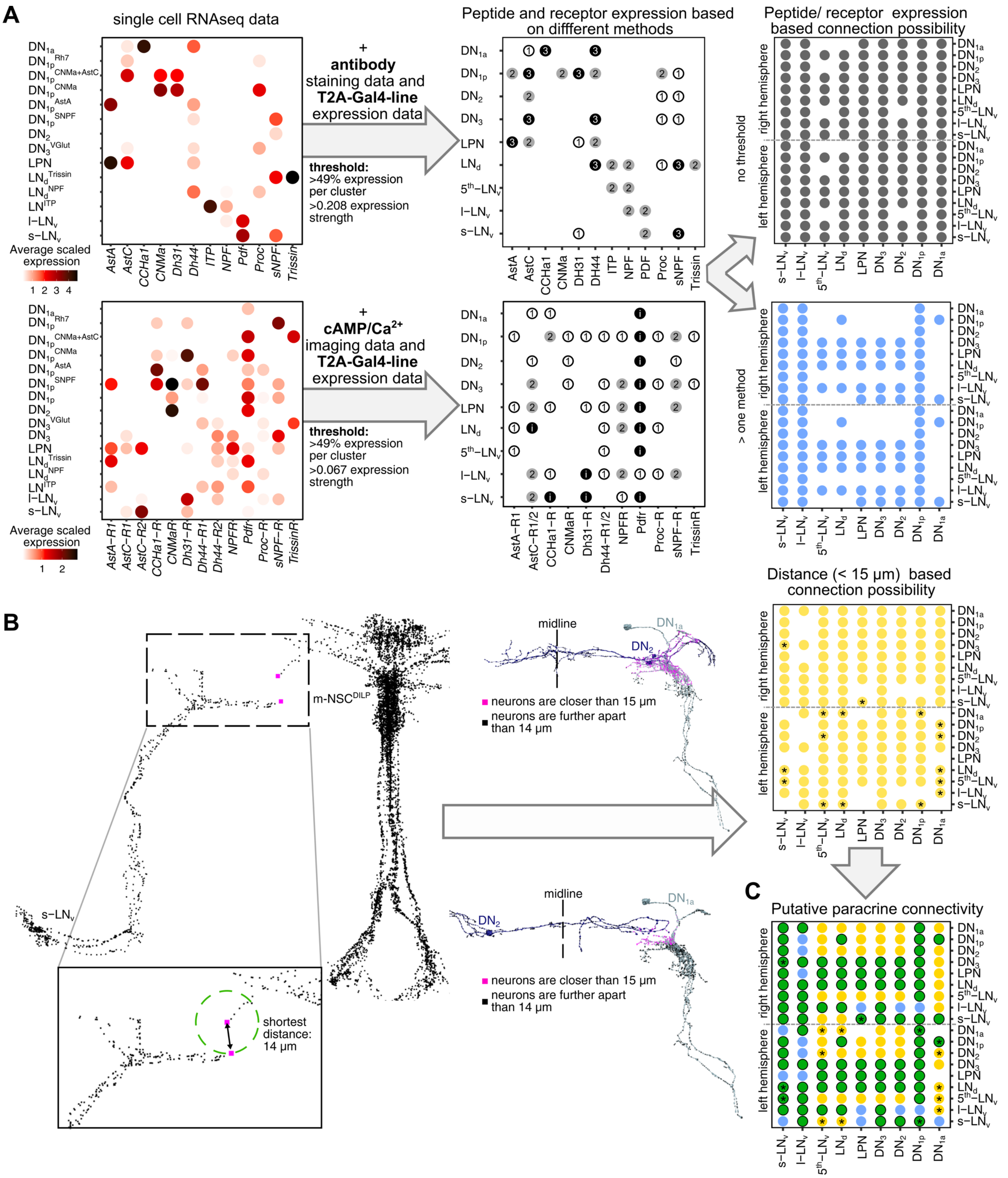

A
single cell RNAseq data
Peptide and receptor expression based on diffferent methods
Peptide/ receptor expression based connection possibility
+ antibody staining data and T2A-Gal4-line expression data
threshold: >49% expression per cluster >0.208 expression strength
+ cAMP/Ca2+ imaging data and T2A-Gal4-line expression data
threshold: >49% expression per cluster >0.067 expression strength
Average scaled expression
no threshold
> one method
right hemisphere
left hemisphere
B
m-NSC DILP
s-LNv
shortest distance: 14 µm
midline
DN1a
DN2
neurons are closer than 15 µm
neurons are further apart than 14 µm
Distance (< 15 µm) based connection possibility
C
Putative paracrine connectivity

Supplementary Fig. 16: Workflow to determine putative paracrine connectivity within the clock network. (A) Expression patterns reported here and in previous studies (see Source Data for this Figure) were used to determine a threshold for the single-cell RNA sequencing data (presented in Figures 6 and 7). 0.208 averaged scaled expression for neuropeptides and 0.067 for receptors was used as a threshold. Additionally, expression in less than 50% of the cells in a given cluster was considered absent. This RNA expression was combined with T2A-Gal4 expression data (Supplementary Fig. 15, Supplementary Data 3 and 4, Figure 7) and antibody or imaging data (i) (see Source Data for this Figure), to generate a peptide/receptor expression map which also takes into account the number of methods that report positive expression. The peptide and receptor expression maps were used to compute a connectivity map. In the absence of any thresholding, all clock clusters communicate with almost all the other clusters via paracrine signaling. To reduce the number of false positives, we only considered expression of peptides and receptors when shown by two independent methods. This filtering reduces the number of putative paracrine connections to some extent (blue). **(B)** The shortest distance between s-LN_v and m-NSC^{DILP} was used as the distance threshold. As shown previously, PDF from the s-LN_v can signal to the m-NSC^{DILP} [8]. For all neuron groups, the distance of the neurons to other neuron groups was calculated using dotprops files of the neuron skeletons. If the distance between them was below the threshold of 15µm, these neurons were considered to be in the range for paracrine signaling (yellow). We also used a highly stringent distance threshold of 1µm to determine putative paracrine connections (asterisks denote connections which do not meet this threshold). **(C)** Combining the expression and distance matrices allows the calculation of a hypothetical connection matrix for paracrine connectivity (green). Connections marked by asterisks do not meet the peptide diffusion distance threshold of 1µm. ~92% of the connections predicted based on the 15µm distance threshold are retained even with this stringent distance threshold. Connections are shown for neurons in the right hemisphere. Source data for panels A and B are provided in the Source Data file.

Supplementary Table 1: Fly strains used in this study

Fly strain	BDRC Stock number / Reference
QUAS-nlsRFP; UAS-trans-Tango	[9]
w; R43D05AD/Cyo; R93B11 DBD/TM6B (DN_{1a})	[10]
yw; + ; Clk4.1M-Gal4 (DN_{1p})	[11]
w; R43D05AD/Cyo; VT003234DBD/TM6B (DN_2)	[10]
w; R67F03AD/Cyo; R77H08DBD/TM6B (DN_3)	70786, 69630 [12]
w; R11B03AD/Cyo; R65D05DBD/TM6B (LPN)	[10]
w; R22E04AD ; R18F07DBD (LN^{ITP})	49309, 70047 (This study)
yw; PDF-Gal4 (LN_v^{PDF})	[13]
w^{1118}	5905
w; +; 10xUAS-myr::GFP (myrGFP)	[14]
UAS-GFP S65T	1522
13×LexAop2-6×GFP	52266
JFRC81-10xUAS-IVS-Syn21-GFP-p10 (EGFP)	[15]
MCFO7	64091 [16]
Clk.9M-Gal4; pdf-Gal80	[17]
tim-(UAS)-Gal4	80941 [18]
CCHa1-LexA	84361
Proc-LexA	84432
AstA-Gal4	84593
AstC-Gal4	84595
CNMa-Gal4	84619
ChAT-Gal4	84618
Dh31-RA/C-Gal4	84623
Dh44-Gal4	84627
sNPF-Gal4	84706
AstC-R1-Gal4	84596
CNMaR-Gal4	84620
Dh31R-RA/B/C-Gal4	84625
Dh31R-RC-Gal4	84626
NPFR-RA/C-Gal4	84672
NPFR-RB/D-Gal4	84673
PDFR-RA-Gal4	84684
PDFR-RA-Gal4	[19]
sNPFR-Gal4	84691
AstC-R2-RB-Gal4	[19]
CCHa1R-Gal4	[19]
VGlut-Gal4	84697

Supplementary Table 2: Antibodies used for immunohistochemistry

Antibody	Dilution	Source / Reference
chicken anti-GFP	1:1000	Rockland, Limerick, PA, USA
chicken anti-GFP	1:1000	Abcam, RRID: AB_300798
guinea pig anti-RFP	1:5000	Gift from Dr. Susan Morton
mouse C7 anti-PDF	1:1000	Developmental Studies Hybridoma Bank; [20]
rabbit anti-ITP	1:5000	[21]
guinea pig anti-ITP	1:1000	[22]
rabbit anti-DH44	1:1000	[23]
rabbit anti-PDP1	1:9000	[24]
rat anti-TIM	1:3000	[25]
mouse nc82 anti-Bruchpilot	1:50	[26] RRID: AB_2314866
mouse M2 anti-FLAG-tag	1:500	Sigma-Aldrich #F1804
human anti V5-tag [SV5-P-K]	1:500	Abcam #ab206562
rabbit anti-HA-tag	1:800	Sigma-Aldrich #H6908
guinea pig anti-VRI	1:2000	[27]
goat dN-19 anti-PER	1:200	Santa Cruz Biotechnology, Cat# sc-15720; [28]; RRID: AB_654018
rabbit anti-PER	1:1000	[29] RRID: AB_2315105
rabbit anti-AstC	1:250	[30] RRID: AB_2569126
goat anti-chicken Alexa Fluor® 488	1:500 to 1:200	Life technologies, Carlsbad, CA, USA; Thermo Fisher Scientific
goat anti-mouse Alexa Fluor® 647	1:500	Life technologies, Carlsbad, CA, USA
goat anti-rabbit Cy3	1:500	Millipore, Billerica, MA, USA
goat anti-rat Cy3	1:500	Millipore, Billerica, MA, USA
goat anti-guinea pig Alexa Fluor® 647	1:400	Thermo Fisher Scientific
goat anti-rabbit Alexa Fluor® 635	1:400	Thermo Fisher Scientific
goat anti-mouse Alexa Fluor® 555	1:400	Thermo Fisher Scientific
goat anti-human F(ab')2 Alexa Fluor® 488	1:200	Thermo Fisher Scientific
donkey anti-guinea pig Alexa Fluor ® 555	1:400	Thermo Fisher Scientific
donkey anti-rabbit Alexa Fluor ® 555	1:400	Thermo Fisher Scientific
donkey anti-goat Alexa Fluor ® 488	1:400	Thermo Fisher Scientific
donkey anti-rabbit Alexa Fluor ® 647	1:400	Thermo Fisher Scientific
donkey anti-mouse Alexa Fluor ® 555	1:200	Thermo Fisher Scientific

Supplemental references

1. Lamaze A, Kratschmer P, Chen KF, Lowe S, Jepson JEC. A Wake-Promoting Circadian Output Circuit in *Drosophila*. *Curr Biol* **28**, 3098-3105 e3093 (2018).

2. Reinhard N*, et al.* The lateral posterior clock neurons of *Drosophila melanogaster* express three neuropeptides and have multiple connections within the circadian clock network and beyond. *J Comp Neurol* **530**, 1507-1529 (2022).

3. Reinhard N*, et al.* The Neuronal Circuit of the Dorsal Circadian Clock Neurons in *Drosophila melanogaster*. *Front Physiol* **13**, 886432 (2022).

4. Schubert FK, Hagedorn N, Yoshii T, Helfrich-Förster C, Rieger D. Neuroanatomical details of the lateral neurons of *Drosophila melanogaster* support their functional role in the circadian system. *J Comp Neurol* **526**, 1209-1231 (2018).

5. Sun L, Jiang RH, Ye WJ, Rosbash M, Guo F. Recurrent circadian circuitry regulates central brain activity to maintain sleep. *Neuron* **110**, 2139-2154 e2135 (2022).

6. Schlegel P*, et al.* Whole-brain annotation and multi-connectome cell typing of *Drosophila*. *Nature* **634**, 139-152 (2024).

7. Mu S*, et al.* 3D reconstruction of cell nuclei in a full *Drosophila* brain. *bioRxiv*, 2021.2011.2004.467197 (2021).

8. Nagy D*, et al.* Peptidergic signaling from clock neurons regulates reproductive dormancy in *Drosophila melanogaster*. *PLoS Genet* **15**, e1008158 (2019).

9. Snell NJ, Fisher JD, Hartmann GG, Zolyomi B, Talay M, Barnea G. Complex representation of taste quality by second-order gustatory neurons in *Drosophila*. *Curr Biol* **32**, 3758-3772 e3754 (2022).

10. Sekiguchi M, Inoue K, Yang T, Luo DG, Yoshii T. A Catalog of GAL4 Drivers for Labeling and Manipulating Circadian Clock Neurons in *Drosophila melanogaster*. *J Biol Rhythms* **35**, 207-213 (2020).

11. Zhang Y, Liu Y, Bilodeau-Wentworth D, Hardin PE, Emery P. Light and temperature control the contribution of specific DN1 neurons to *Drosophila* circadian behavior. *Curr Biol* **20**, 600-605 (2010).

12. Meiselman MR*, et al.* Recovery from cold-induced reproductive dormancy is regulated by temperature-dependent AstC signaling. *Curr Biol* **32**, 1362-1375 e1368 (2022).

13. Park JH, Helfrich-Förster C, Lee G, Liu L, Rosbash M, Hall JC. Differential regulation of circadian pacemaker output by separate clock genes in *Drosophila*. *Proc Natl Acad Sci U S A* **97**, 3608-3613 (2000).

14. Pfeiffer BD*, et al.* Refinement of tools for targeted gene expression in *Drosophila*. *Genetics* **186**, 735-755 (2010).

15. Pfeiffer BD, Truman JW, Rubin GM. Using translational enhancers to increase transgene expression in *Drosophila*. *Proc Natl Acad Sci U S A* **109**, 6626-6631 (2012).

16. Nern A, Pfeiffer BD, Rubin GM. Optimized tools for multicolor stochastic labeling reveal diverse stereotyped cell arrangements in the fly visual system. *Proc Natl Acad Sci U S A* **112**, E2967-2976 (2015).

17. Kaneko H*, et al.* Circadian rhythm of temperature preference and its neural control in *Drosophila*. *Curr Biol* **22**, 1851-1857 (2012).

18. Blau J, Young MW. Cycling vrille expression is required for a functional *Drosophila* clock. *Cell* **99**, 661-671 (1999).

19. Kondo S*, et al.* Neurochemical Organization of the *Drosophila* Brain Visualized by Endogenously Tagged Neurotransmitter Receptors. *Cell Rep* **30**, 284-297 e285 (2020).

20. Cyran SA, Yiannoulos G, Buchsbaum AM, Saez L, Young MW, Blau J. The double-time protein kinase regulates the subcellular localization of the *Drosophila* clock protein period. *J Neurosci* **25**, 5430-5437 (2005).

21. Hermann-Luibl C, Yoshii T, Senthilan PR, Dircksen H, Helfrich-Forster C. The ion transport peptide is a new functional clock neuropeptide in the fruit fly *Drosophila melanogaster*. *J Neurosci* **34**, 9522-9536 (2014).

22. Manoli G, Zandawala M, Yoshii T, Helfrich-Förster C. Characterization of clock-related proteins and neuropeptides in *Drosophila littoralis* and their putative role in diapause. *J Comp Neurol*, (2023).

23. Cabrero P*, et al.* The Dh gene of *Drosophila melanogaster* encodes a diuretic peptide that acts through cyclic AMP. *J Exp Biol* **205**, 3799-3807 (2002).

24. Cyran SA*, et al.* vrille, Pdp1, and dClock form a second feedback loop in the *Drosophila* circadian clock. *Cell* **112**, 329-341 (2003).

25. Yoshii T, Todo T, Wulbeck C, Stanewsky R, Helfrich-Forster C. Cryptochrome is present in the compound eyes and a subset of *Drosophila's* clock neurons. *J Comp Neurol* **508**, 952-966 (2008).

26. Wagh DA*, et al.* Bruchpilot, a protein with homology to ELKS/CAST, is required for structural integrity and function of synaptic active zones in *Drosophila*. *Neuron* **49**, 833-844 (2006).

27. Glossop NR, Houl JH, Zheng H, Ng FS, Dudek SM, Hardin PE. VRILLE feeds back to control circadian transcription of Clock in the *Drosophila* circadian oscillator. *Neuron* **37**, 249-261 (2003).

28. Shiga S, Numata H. Roles of PER immunoreactive neurons in circadian rhythms and photoperiodism in the blow fly, *Protophormia terraenovae*. *J Exp Biol* **212**, 867-877 (2009).

29. Stanewsky R, Frisch B, Brandes C, Hamblen-Coyle MJ, Rosbash M, Hall JC. Temporal and spatial expression patterns of transgenes containing increasing amounts of the *Drosophila* clock gene period and a lacZ reporter: mapping elements of the PER protein involved in circadian cycling. *J Neurosci* **17**, 676-696 (1997).

30. Park D, Veenstra JA, Park JH, Taghert PH. Mapping peptidergic cells in *Drosophila*: where DIMM fits in. *PLoS One* **3**, e1896 (2008).
