# Supplementary material for: Synaptic connectome of the Drosophila circadian clock
Source: Nat Commun. 2024 Dec 5;15:10392. doi: 10.1038/s41467-024-54694-0 (PMC11621569; doi:10.1038/s41467-024-54694-0)
Supplement: Supplementary file 2 — Description of Additional Supplementary Files [file 41467_2024_54694_MOESM2_ESM.pdf]

Description of Additional Supplementary Files

File name: Supplementary Data 1
Description: FlyWire cell IDs of identified clock neurons.

File name: Supplementary Data 2
Description: Hemibrain cell IDs of identified clock neurons.

File name: Supplementary Data 3
Description: Neuropeptide expression in clock neurons. Confocal stacks showing expression of GFP (specifically in clock neurons) driven by different neuropeptide-T2A-Gal4 lines. Arrowheads indicate GFP-expressing clock neurons. For all Gal4 lines, panel **(A)** shows a brain overview, and subsequent panels show detailed images of clock neurons. Scale bars = 100µm for overview and 20µm for detail images. Abbreviations: TIM, Timeless; PDF, Pigment dispersing factor; PER, Period.

File name: Supplementary Data 4
Description: Neuropeptide receptor expression in clock neurons. Confocal stacks showing expression of GFP (specifically in clock neurons) driven by different neuropeptide receptor-T2A-Gal4 lines. Arrowheads indicate GFP-expressing clock neurons. For all Gal4 lines, panel **(A)** shows a brain overview, and subsequent panels show detailed images of clock neurons. Scale bars = 100µm for overview and 20µm for detail images. Abbreviations: TIM, Timeless; PDF, Pigment dispersing factor.

File name: Supplementary Movie 1
Description: Reconstructions of single clock neurons from the FlyWire dataset displayed by cluster.

File name: Supplementary Movie 2
Description: Reconstructions of all clock neurons from the FlyWire dataset.

File name: Supplementary Movie 3
Description: All DN_3 are labeled by *tim-(UAS)-Gal4* and could be counted using antibody staining against Period (PER) and Vrille (VRI).
