# Supplementary material for: Synaptic connectome of the Drosophila circadian clock
Source: Nat Commun. 2024 Dec 5;15:10392. doi: 10.1038/s41467-024-54694-0 (PMC11621569; doi:10.1038/s41467-024-54694-0)

Supplementary Data 3: Neuropeptide expression in clock neurons. Confocal stacks showing expression of GFP (specifically in clock neurons) driven by different neuropeptide-T2A-Gal4 lines. Arrow heads indicate GFP-expressing clock neurons. For all Gal4 lines, panel A shows brain overview, and subsequent panels show detail images of clock neurons. Scale bars = 100 µm for overview and 20 µm for detail images. Abbreviations: TIM, Timeless; PDF, Pigment dispersing factor; PER, Period.

AstA-Gal4

DN_{1p}, LPN

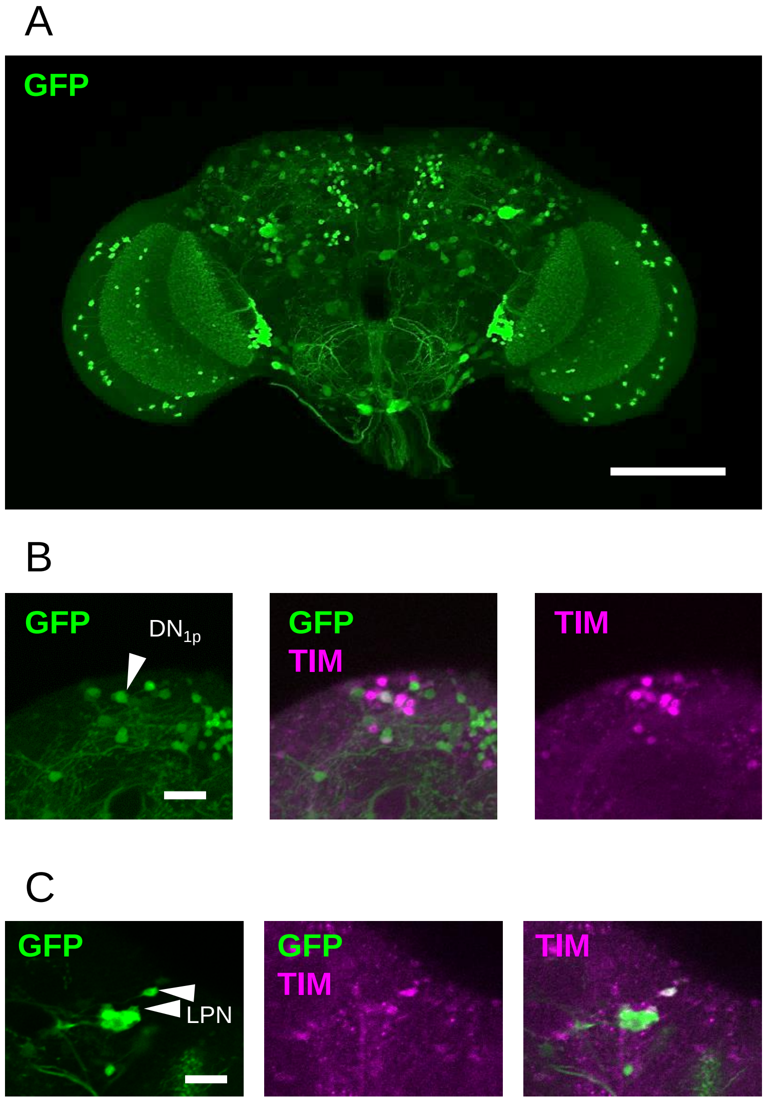

AstC-Gal4

DN_{1p}, DN_2, DN_3, LPN

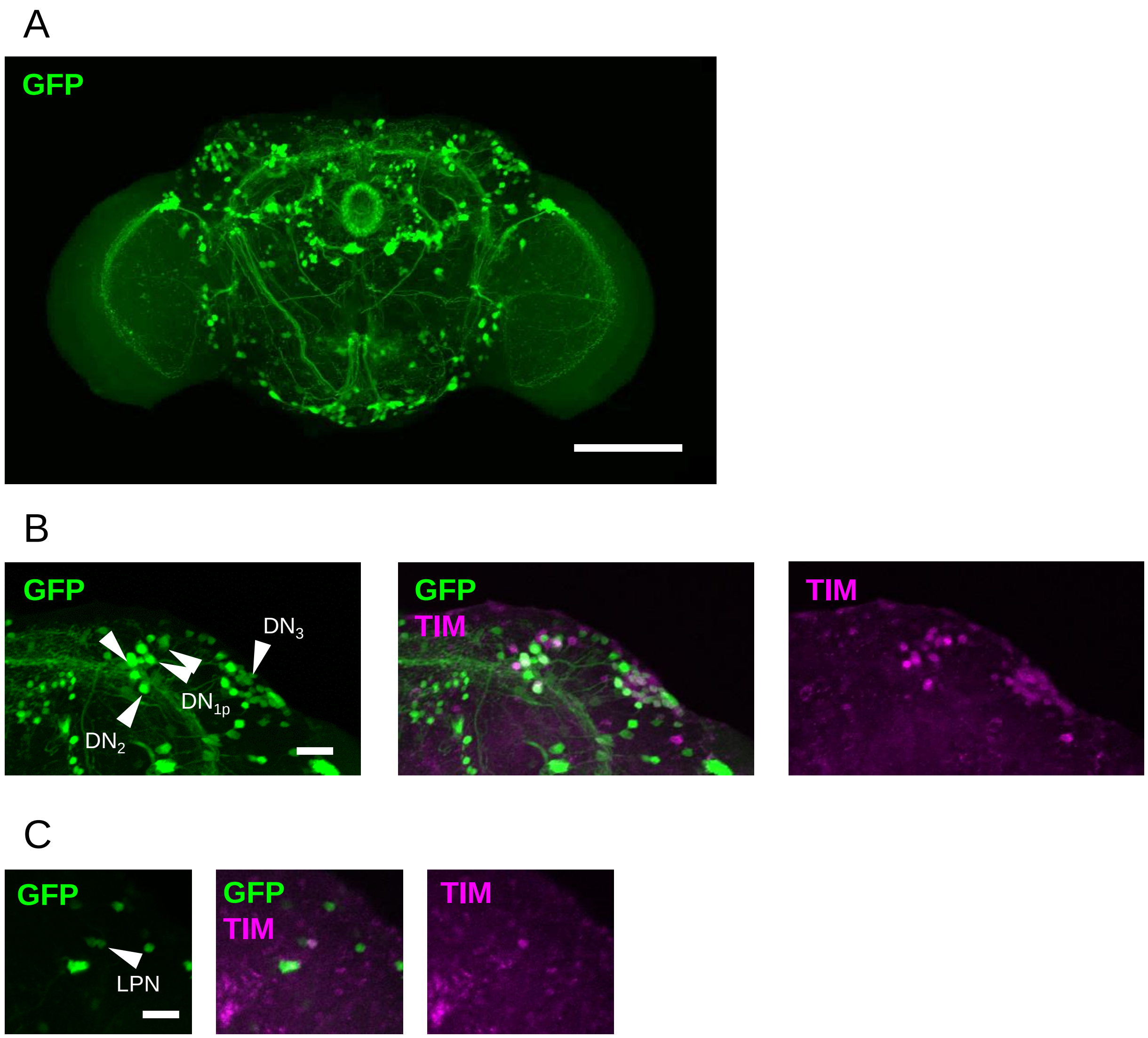

CCHa1-LexA

DN_{1a}

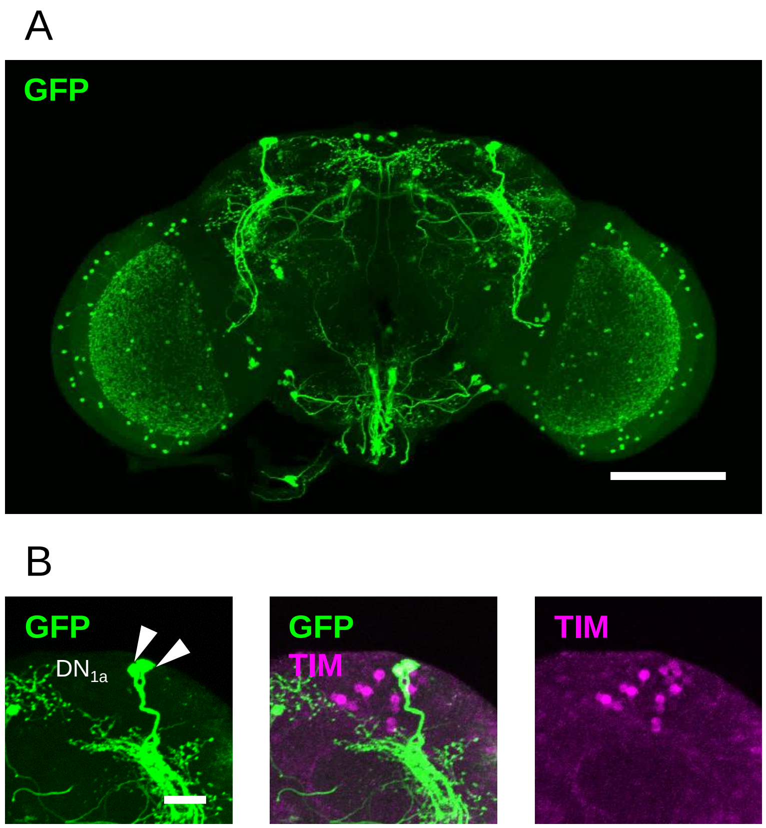

CNMa-Gal4
DN_{1p}

A

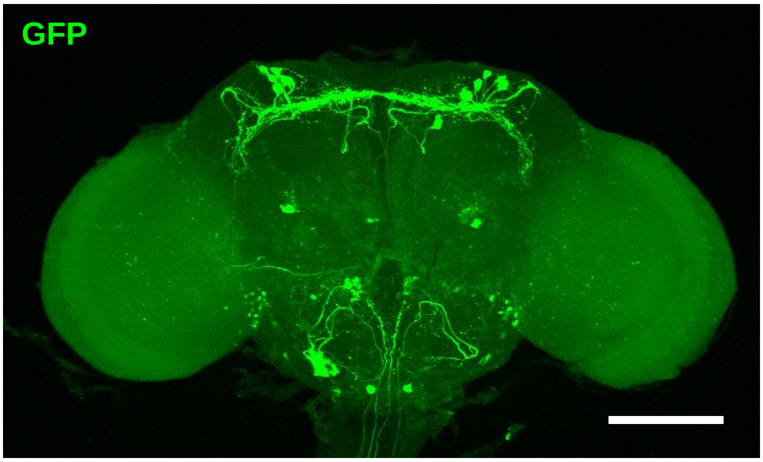

B

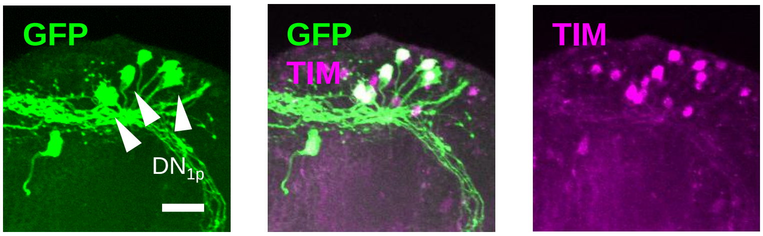

Dh31-RA/C-Gal4

DN_{1p}, s-LN_v

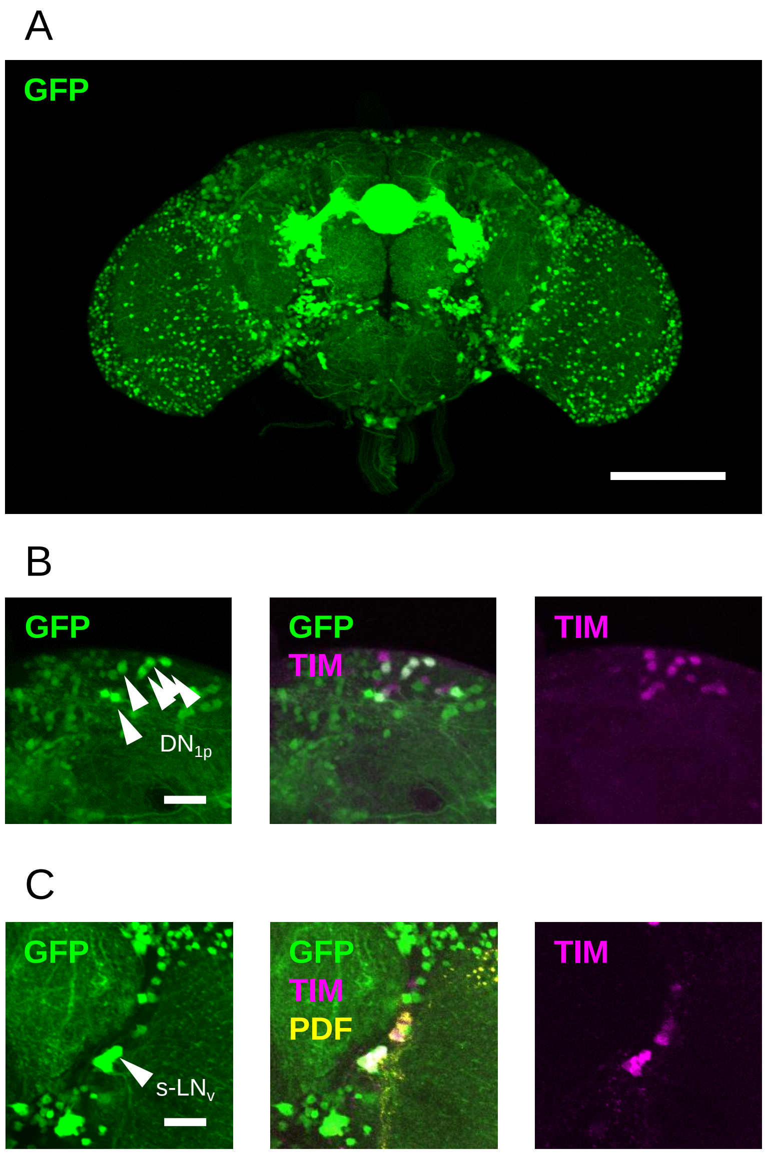

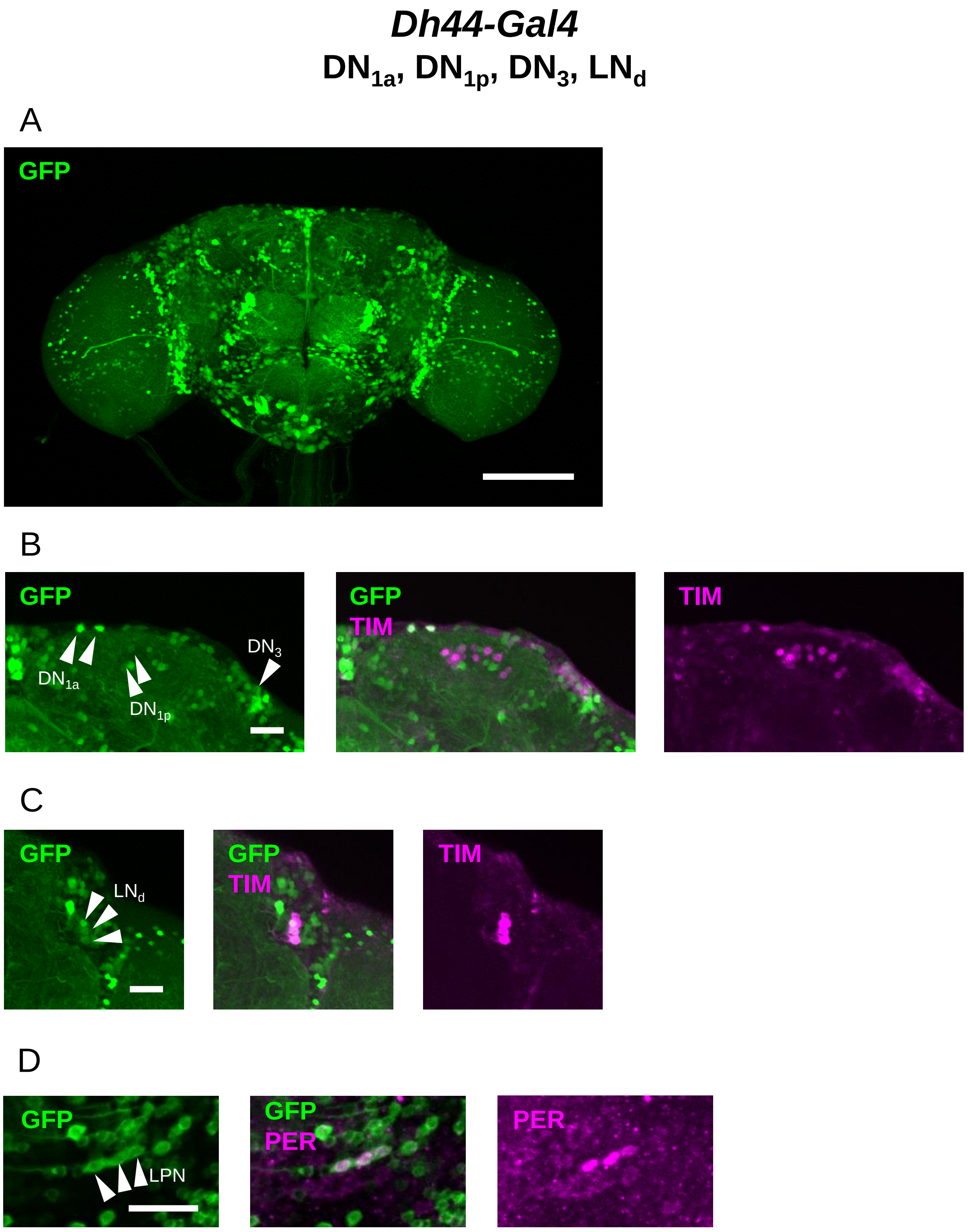

Dh44-Gal4
DN1a, DN1p, DN3, LNd
A
GFP
B
GFP
DN1a
DN1p
DN3
GFP
TIM
TIM
C
GFP
LNd
GFP
TIM
TIM
D
GFP
LPN
GFP
PER
PER

Proc-LexA

DN_{1p}

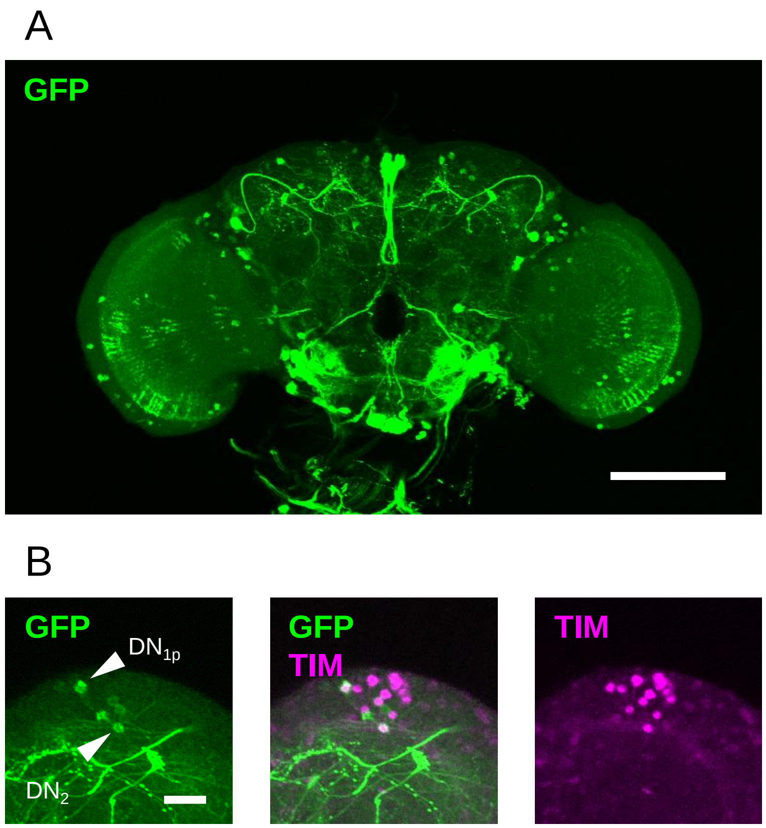

sNPF-Gal4

DN$_3$, LN$_d$, s-LN$_v$

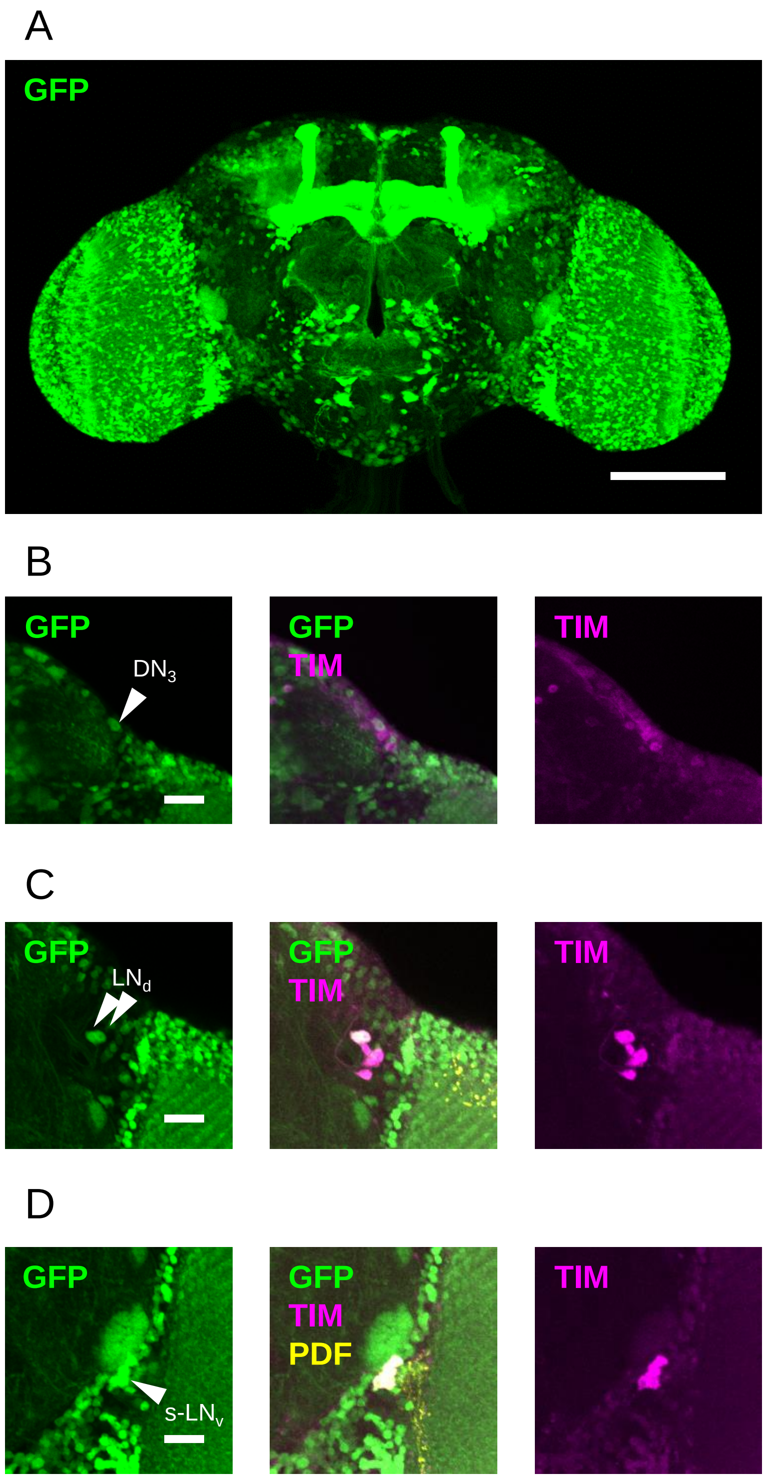

Supplement: Supplementary file 5 — Supplementary Data 3 [file 41467_2024_54694_MOESM5_ESM.pdf]
