# Supplementary material for: Synaptic connectome of the Drosophila circadian clock
Source: Nat Commun. 2024 Dec 5;15:10392. doi: 10.1038/s41467-024-54694-0 (PMC11621569; doi:10.1038/s41467-024-54694-0)

Supplementary Data 4: Neuropeptide receptor expression in clock neurons. Confocal stacks showing expression of GFP (specifically in clock neurons) driven by different neuropeptide receptor-T2A-Gal4 lines. Arrow heads indicate GFP-expressing clock neurons. For all Gal4 lines, panel A shows brain overview, and subsequent panels show detail images of clock neurons. Scale bars = 100 μm for overview and 20 μm for detail images. Abbreviations: TIM, Timeless; PDF, Pigment dispersing factor.

AstC-R1-Gal4

DN_2, DN_3, LPN

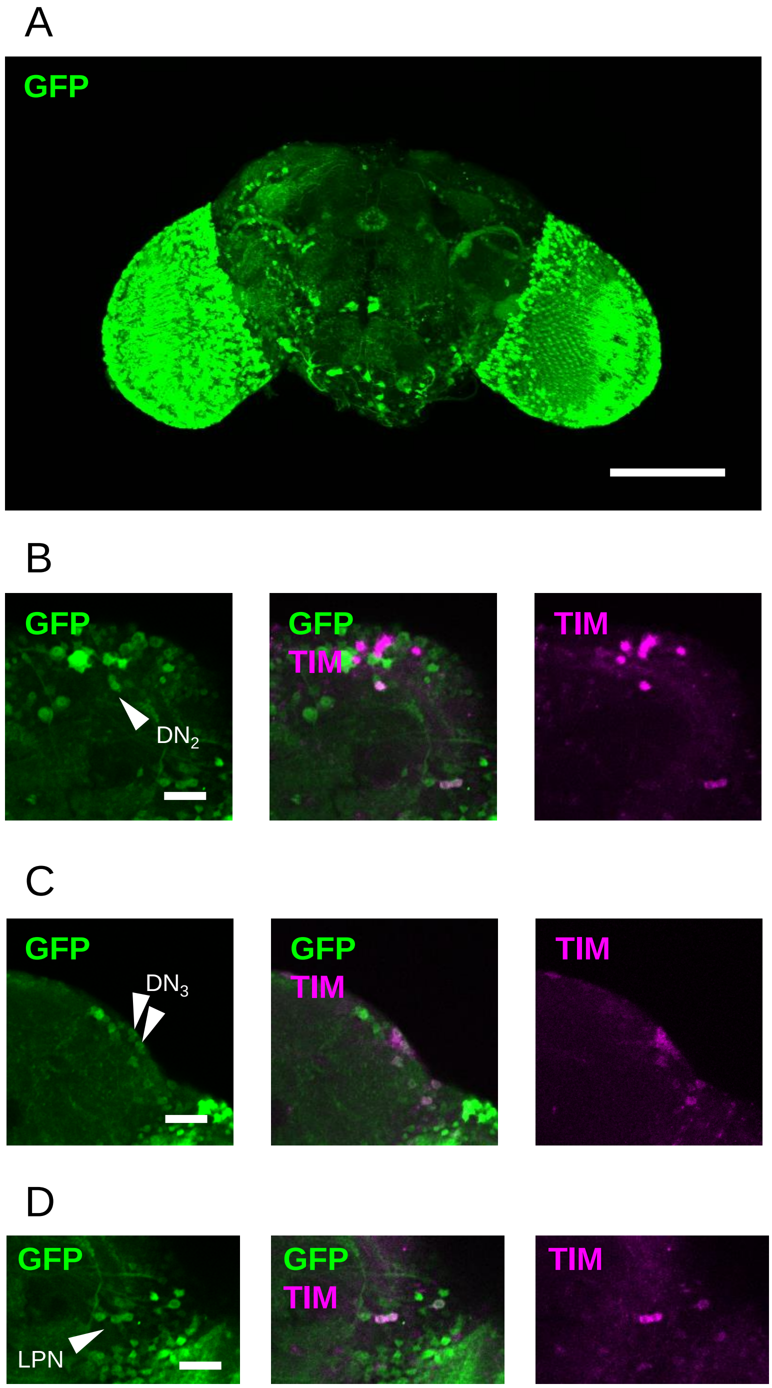

AstC-R2-RB-Gal4

DN_{1a}, DN_2, DN_3, LN_d, s-LN_v, l-LN_v

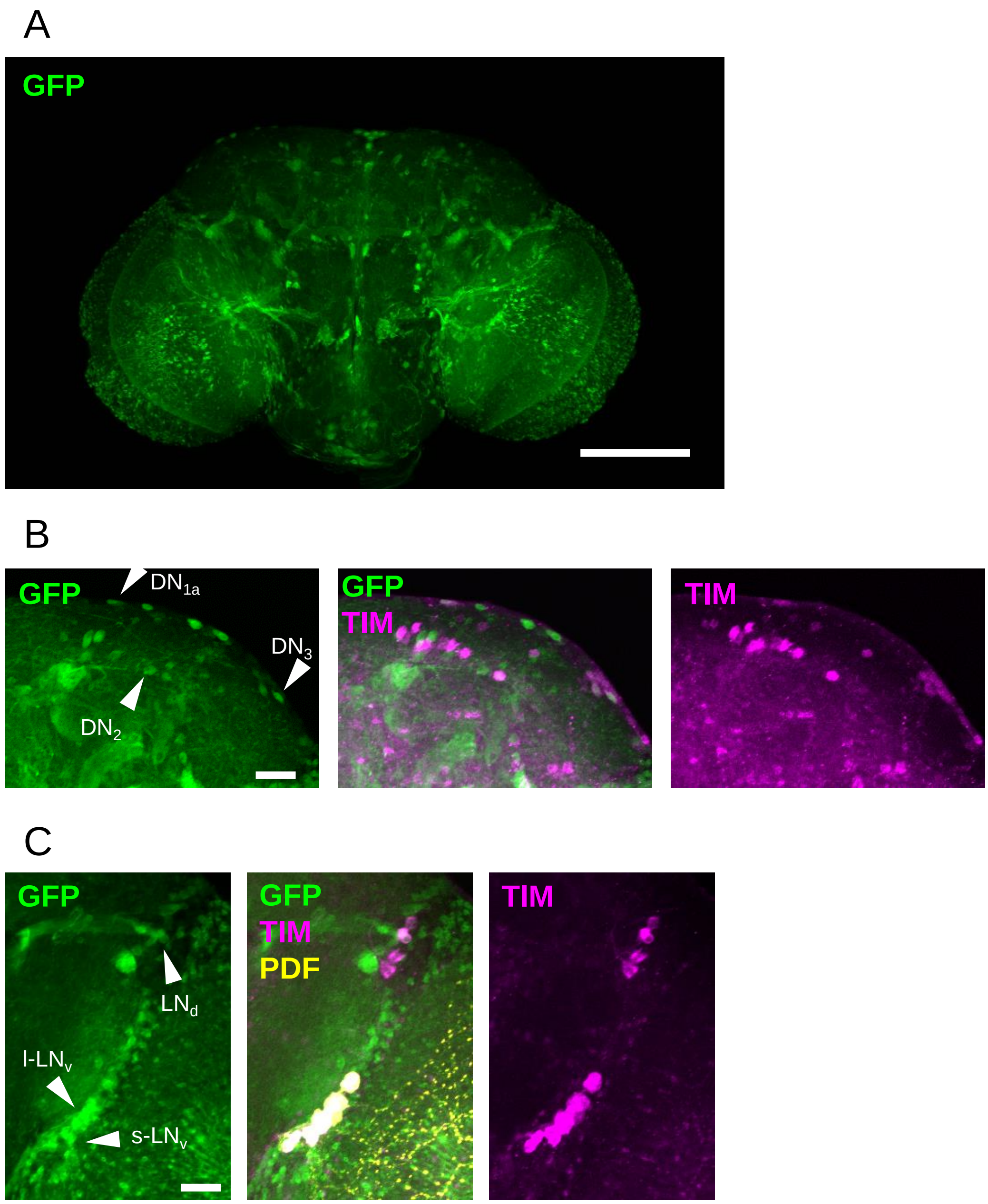

CCHa1R-Gal4

DN_{1a}, DN_{1p}, LPN, s-LN_v, l-LN_v

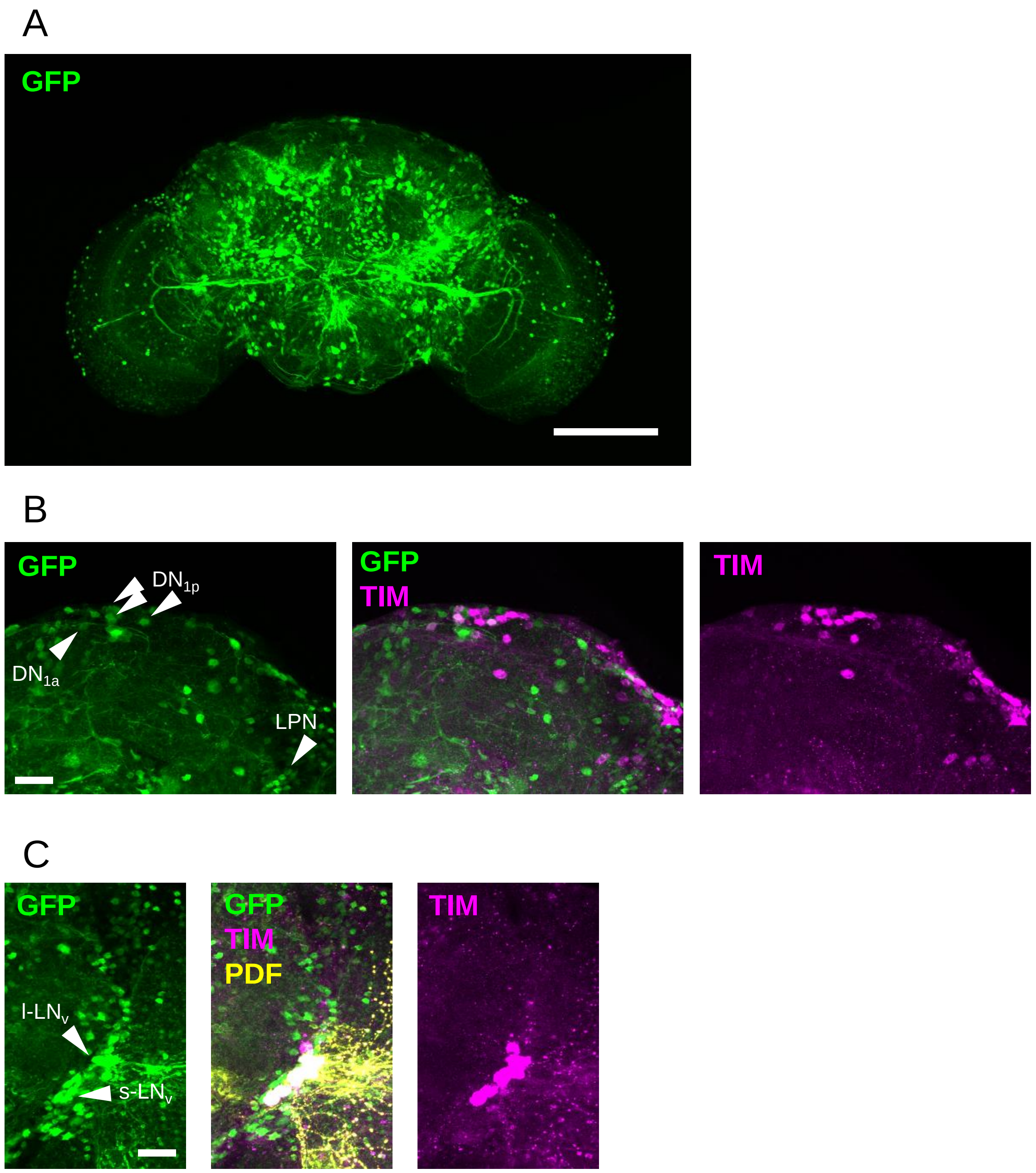

CNMaR-Gal4

DN_3

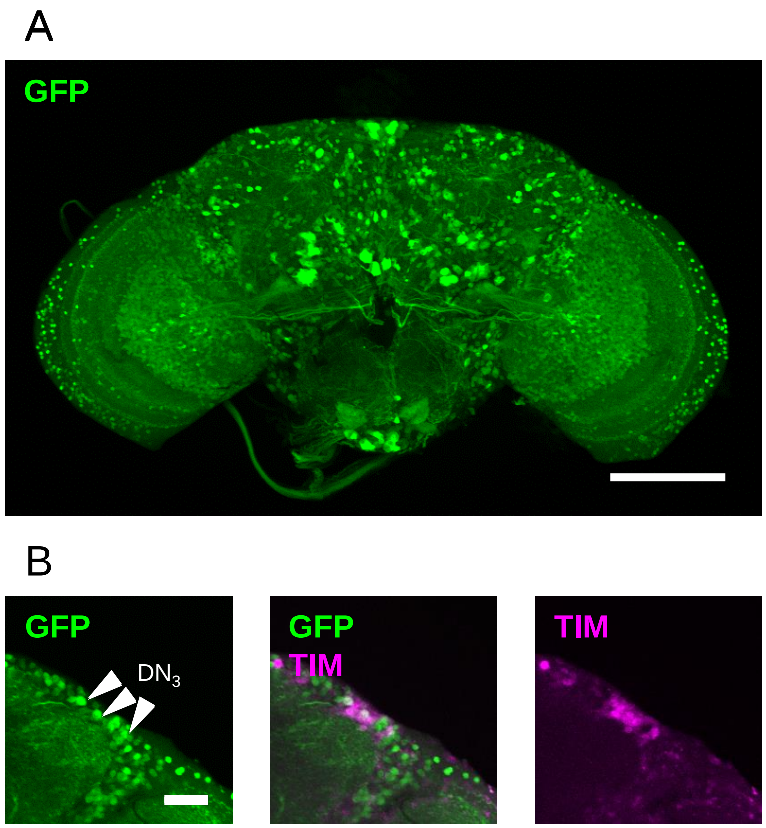

Dh31R-RA/B/C-Gal4

l-LN_v

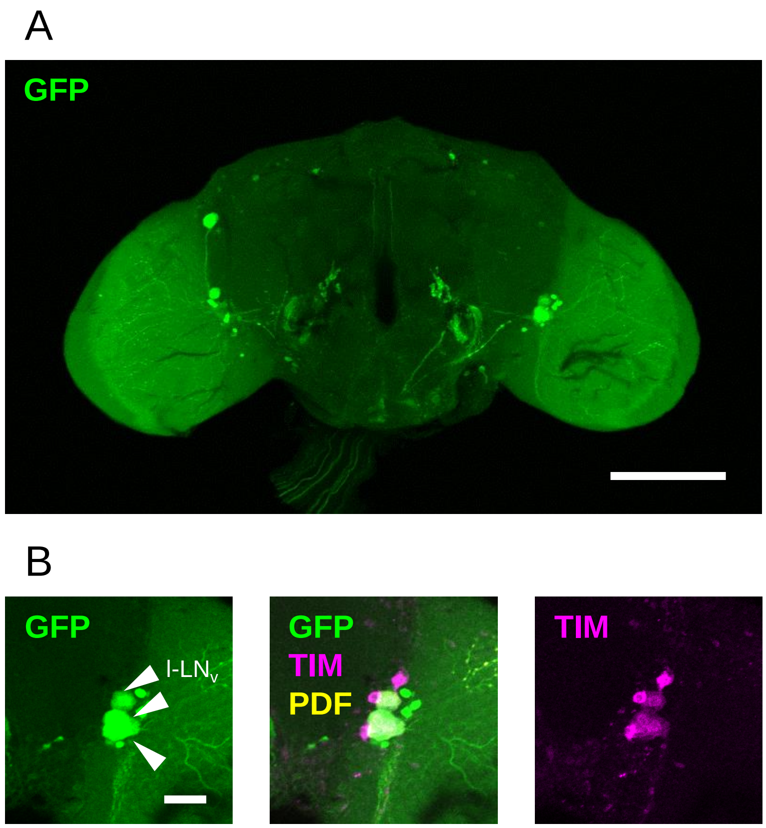

Dh31R-RC-Gal4

l-LN$_v$

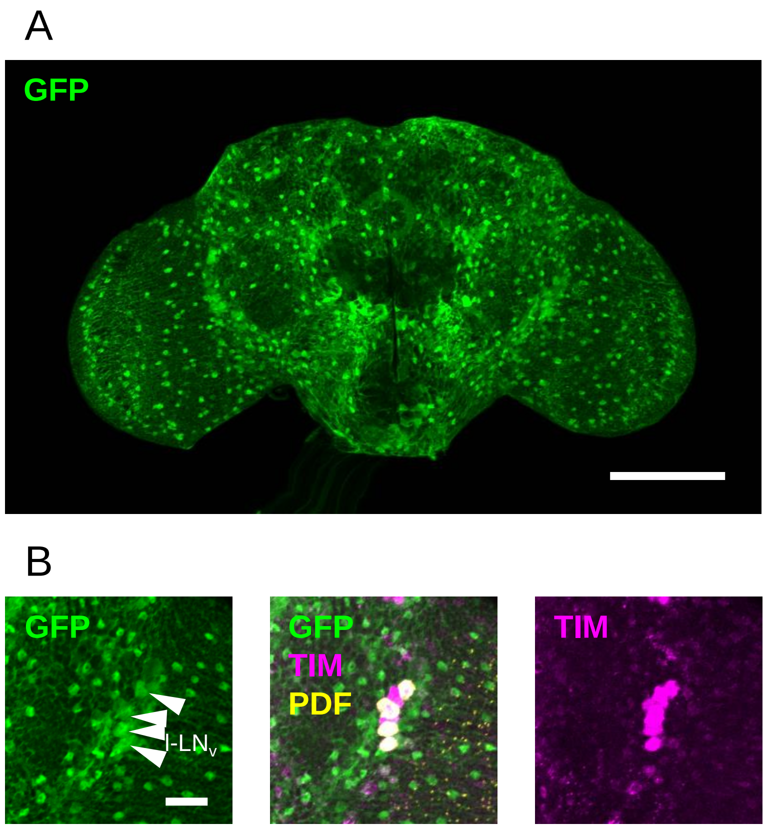

NPFR-RA/C-Gal4

DN_3, LN_d, s-LN_v, l-LN_v

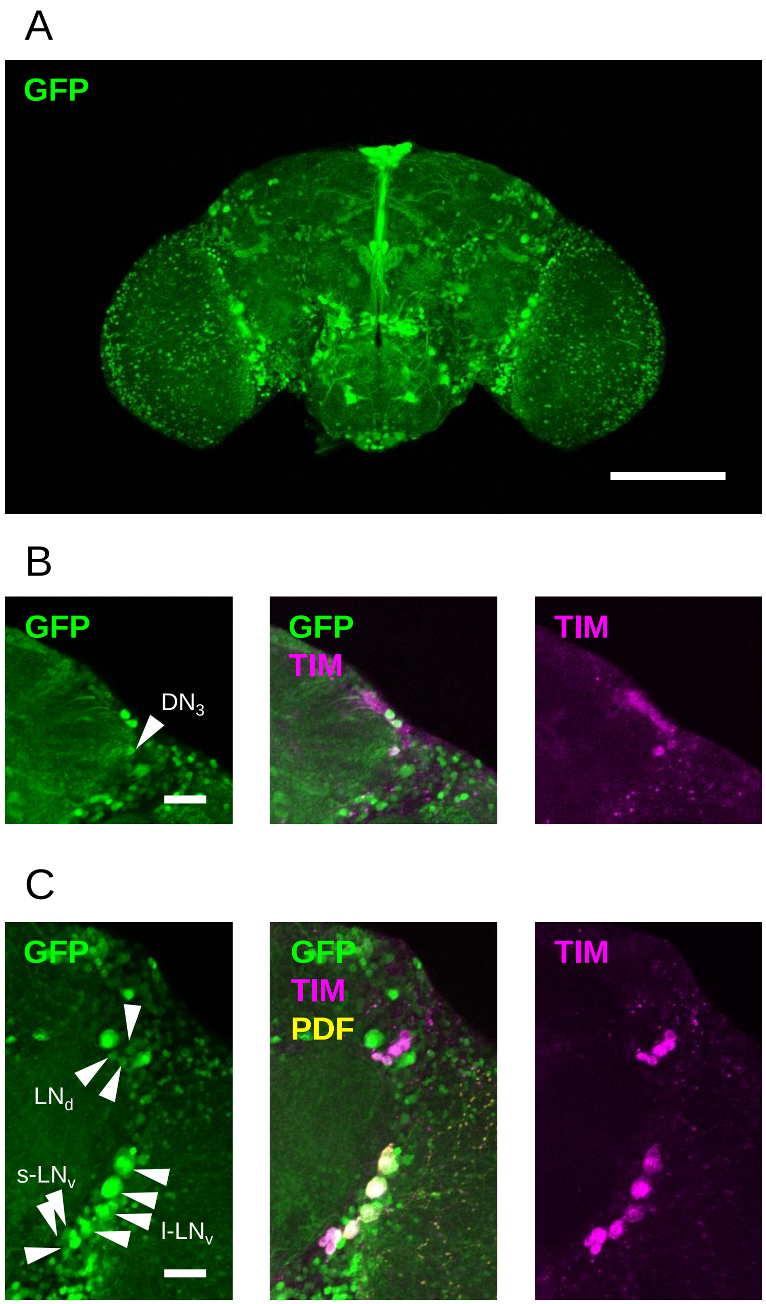

NPFR-RB/D-Gal4

l-LN_v, LPN

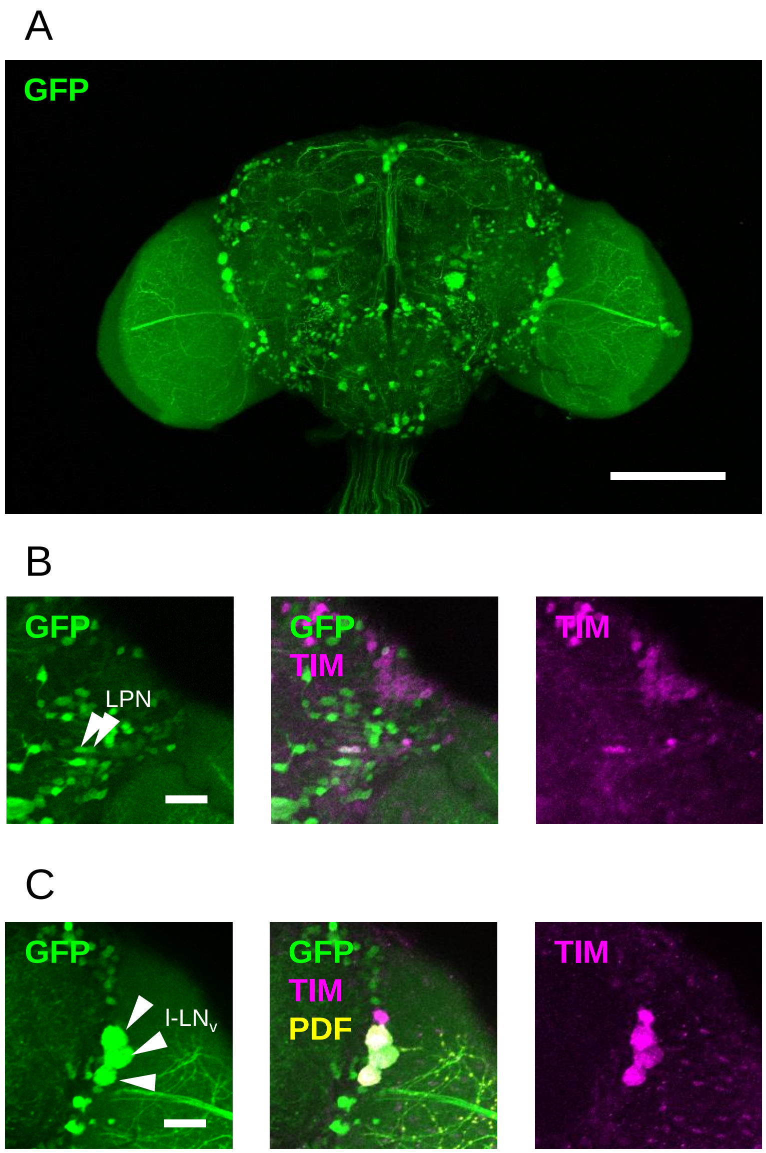

Pdfr-RA-Gal4

DN_{1a}, DN_{1p}, DN_2, DN_3, LPN, LN_d, 5th-LN_v, l-LN_v, s-LN_v

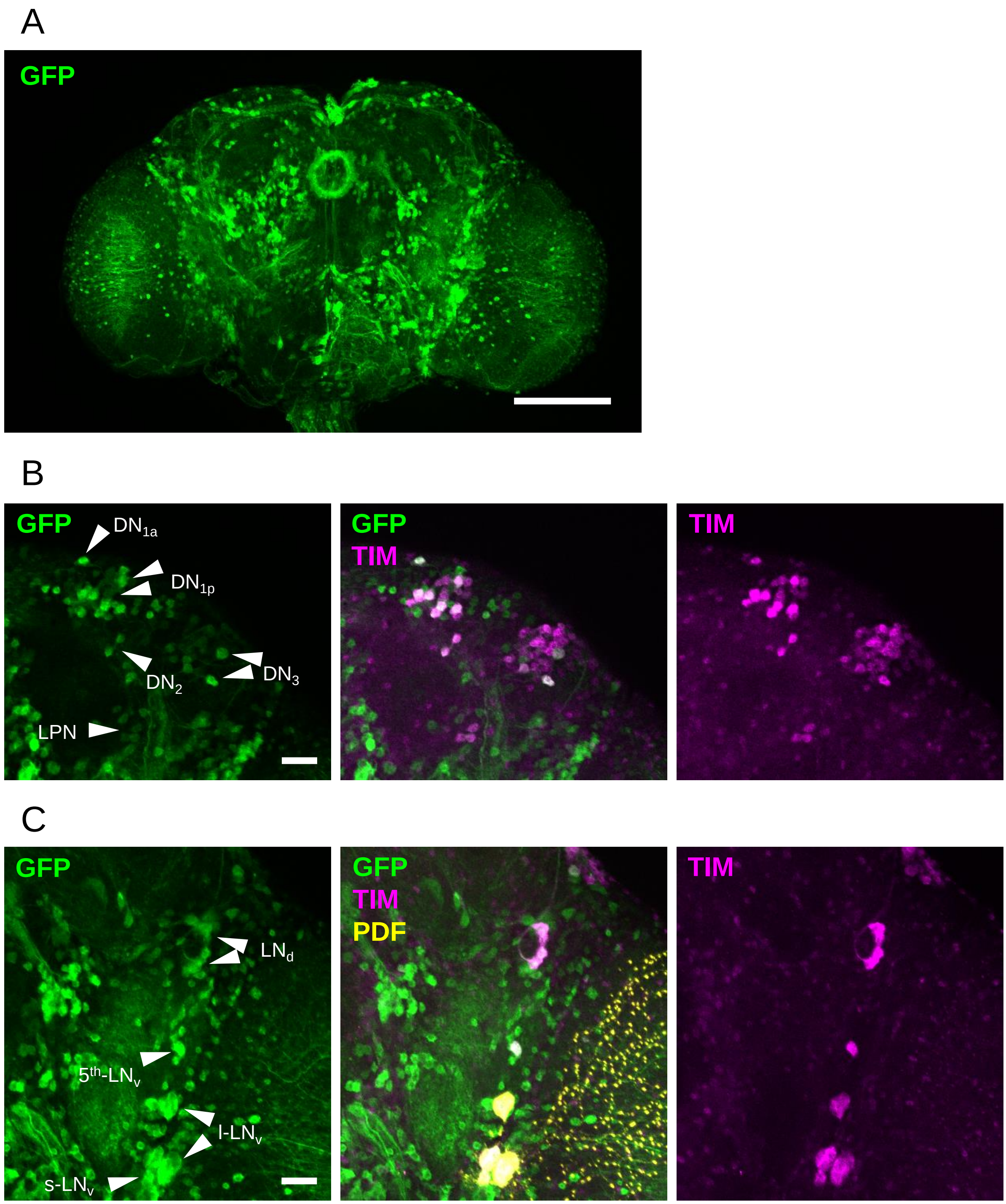

sNPFR-Gal4

DN_{1p} DN_2, DN_3, LPN, l-LN_v

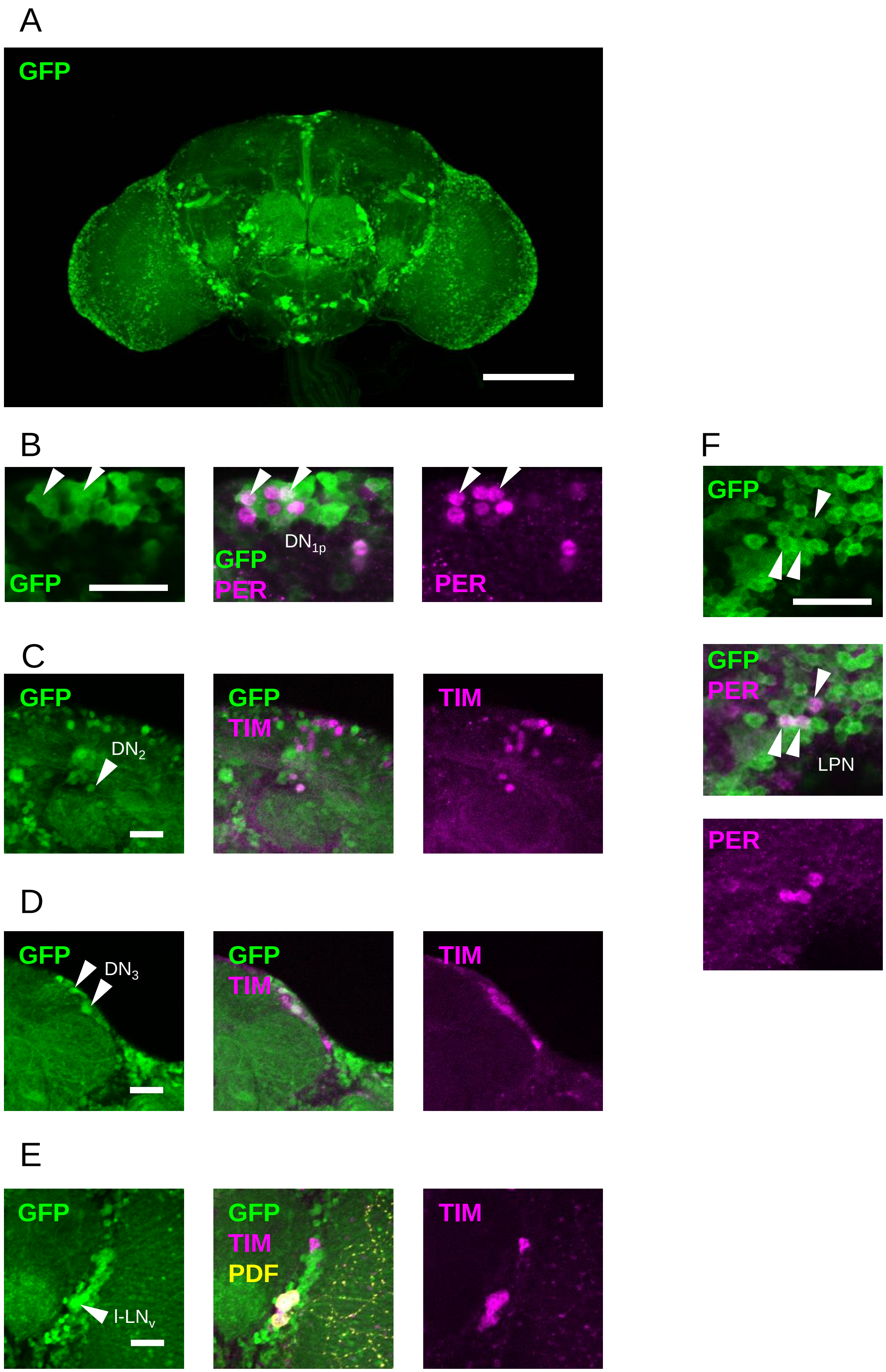

Supplement: Supplementary file 6 — Supplementary Data 4 [file 41467_2024_54694_MOESM6_ESM.pdf]
